# Metabolic alterations in a rat model of takotsubo syndrome

**DOI:** 10.1093/cvr/cvab081

**Published:** 2021-03-12

**Authors:** Nadine Godsman, Michael Kohlhaas, Alexander Nickel, Lesley Cheyne, Marco Mingarelli, Lutz Schweiger, Claire Hepburn, Chantal Munts, Andy Welch, Mirela Delibegovic, Marc Van Bilsen, Christoph Maack, Dana K Dawson

**Affiliations:** Aberdeen Cardiovascular and Diabetes Centre, University of Aberdeen, Polwarth Building, Foresterhill, Aberdeen AB25 2ZD, UK; Comprehensive Heart Failure Center (CHFC), Würzburg, Deutsches Zentrum für Herzinsuffizienz Würzburg, Universitätsklinikum Würzburg, Am Schwarzenberg 15, Haus A15, 97078 Würzburg, Germany; Comprehensive Heart Failure Center (CHFC), Würzburg, Deutsches Zentrum für Herzinsuffizienz Würzburg, Universitätsklinikum Würzburg, Am Schwarzenberg 15, Haus A15, 97078 Würzburg, Germany; Aberdeen Cardiovascular and Diabetes Centre, University of Aberdeen, Polwarth Building, Foresterhill, Aberdeen AB25 2ZD, UK; Biomedical Physics, University of Aberdeen, Aberdeen AB25 2ZD, UK; John Mallard Scottish P.E.T. Centre, University of Aberdeen, Aberdeen AB25 2ZD, UK; Aberdeen Cardiovascular and Diabetes Centre, University of Aberdeen, Polwarth Building, Foresterhill, Aberdeen AB25 2ZD, UK; School for Cardiovascular Diseases, Faculty of Health, Medicine and Life Sciences - Maastricht University, Universiteitssingel 40, 6229 ER Maastricht, Netherlands; Biomedical Physics, University of Aberdeen, Aberdeen AB25 2ZD, UK; Aberdeen Cardiovascular and Diabetes Centre, University of Aberdeen, Polwarth Building, Foresterhill, Aberdeen AB25 2ZD, UK; School for Cardiovascular Diseases, Faculty of Health, Medicine and Life Sciences - Maastricht University, Universiteitssingel 40, 6229 ER Maastricht, Netherlands; Comprehensive Heart Failure Center (CHFC), Würzburg, Deutsches Zentrum für Herzinsuffizienz Würzburg, Universitätsklinikum Würzburg, Am Schwarzenberg 15, Haus A15, 97078 Würzburg, Germany; Aberdeen Cardiovascular and Diabetes Centre, University of Aberdeen, Polwarth Building, Foresterhill, Aberdeen AB25 2ZD, UK

**Keywords:** Takotsubo, Metabolism, Energetics, Inflammation, Remodelling, Heart failure

## Abstract

**Aims:**

Cardiac energetic impairment is a major finding in takotsubo patients. We investigate specific metabolic adaptations to direct future therapies.

**Methods and results:**

An isoprenaline-injection female rat model (vs. sham) was studied at Day 3; recovery assessed at Day 7. Substrate uptake, metabolism, inflammation, and remodelling were investigated by ^18^F-fluorodeoxyglucose (^18^F-FDG) positron emission tomography, metabolomics, quantitative PCR, and western blot (WB). Isolated cardiomyocytes were patch-clamped during stress protocols for redox states of NAD(P)H/FAD or [Ca^2+^]_c_, [Ca^2+^]_m_, and sarcomere length. Mitochondrial respiration was assessed by seahorse/Clark electrode (glycolytic and β-oxidation substrates). Cardiac ^18^F-FDG metabolic rate was increased in takotsubo (*P* = 0.006), as was the expression of GLUT4-RNA/GLUT1/HK2-RNA and HK activity (all *P* < 0.05), with concomitant accumulation of glucose- and fructose-6-phosphates (*P* > 0.0001). Both lactate and pyruvate were lower (*P* < 0.05) despite increases in LDH-RNA and PDH (*P* < 0.05 both). β-Oxidation enzymes CPT1b-RNA and 3-ketoacyl-CoA thiolase were increased (*P* < 0.01) but malonyl-CoA (CPT-1 regulator) was upregulated (*P* = 0.01) with decreased fatty acids and acyl-carnitines levels (*P* = 0.0001–0.02). Krebs cycle intermediates α-ketoglutarate and succinyl-carnitine were reduced (*P* < 0.05) as was cellular ATP reporter dihydroorotate (*P* = 0.003). Mitochondrial Ca^2+^ uptake during high workload was impaired on Day 3 (*P* < 0.0001), inducing the oxidation of NAD(P)H and FAD (*P* = 0.03) but resolved by Day 7. There were no differences in mitochondrial respiratory function, sarcomere shortening, or [Ca^2+^] transients of isolated cardiomyocytes, implying preserved integrity of both mitochondria and cardiomyocyte. Inflammation and remodelling were upregulated—increased CD68-RNA, collagen RNA/protein, and skeletal actin RNA (all *P* < 0.05).

**Conclusion:**

Dysregulation of glucose and lipid metabolic pathways with decreases in final glycolytic and β-oxidation metabolites and reduced availability of Krebs intermediates characterizes takotsubo myocardium. The energetic deficit accompanies defective Ca^2+^ handling, inflammation, and upregulation of remodelling pathways, with the preservation of sarcomeric and mitochondrial integrity.

## Graphical Abstract

**Graphical Abstract:**
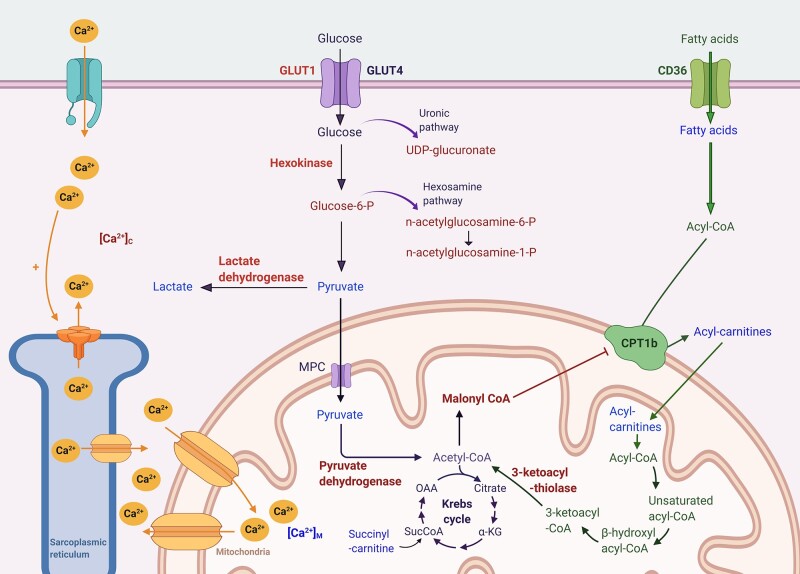

## 1. Introduction

Takotsubo syndrome is an acute heart failure syndrome often triggered by severe emotional stress. The pattern of myocardial dysfunction observed in takotsubo syndrome is unique, as is the course of its subsequent spontaneous recovery. Despite the latter, patients who suffer a prior episode of takotsubo have long-term prognosis similar to patients with myocardial infarction.^1^ It is therefore important to investigate the malfunctioning pathways, in order to address therapy in a logical, disease-specific approach.

We have previously demonstrated an energetic deficit in the acute and convalescent takotsubo human hearts,^2^ which contributes to the chronic heart failure phenotype subsequently developing in a proportion of patients.^3^ The only mechanistic approach to date to explain this energetic impairment was provided by the evidence of an increased nitrosative stress in the takotsubo myocardium.^4,^^5^ Other groups demonstrated abnormalities in myocardial substrate uptake, although reports remain at variance, some showing increased^6,^^7^ or decreased^8,^^9^ myocardial glucose uptake and impaired fatty acid metabolism.^10^

To develop a therapeutic approach for takotsubo syndrome, a deeper understanding of which metabolic, energetic, and mitochondrial alterations underlie this transient form of cardiomyopathy is required. As availability of human myocardial tissue (especially uncomplicated by underlying comorbidities) is limited and timely access to enough such sample would be impractical, we conducted experiments in a previously established takotsubo rat model,^11–13^ investigating respiratory chain function, Ca^2+^ handling, redox regulation, and reactive oxygen species formation in both isolated mitochondria and when integrated into their physiological environment in cardiac myocytes. Furthermore, we explored sarcomere shortening, gene and protein expression of glucose and fatty acids metabolic pathways, the myocardial metabolomic profile, and examined myocardial substrate utilization *in vivo* by ^18^F-fluorodeoxyglucose (^18^F-FDG) positron emission tomography (PET).

## 2. Methods

### 2.1 Animal studies

All study procedures complied with the United Kingdom Home Office Regulations for use of laboratory animals and were carried out in accordance with the Animals (Scientific Procedures) Act 1986 and the Directive 2010/63/EU of the European Parliament on the protection of animals used for scientific purposes. All studies were approved by the University of Aberdeen Ethics Committee.

### 2.2 In vivo rat model of takotsubo syndrome

Female Sprague-Dawley rats (2–4 months of age, 241 ± 55 g) underwent a single intraperitoneal injection of 100 mg/kg isoprenaline (Sigma) to induce takotsubo left ventricular (LV) dysfunction as previously described^11,^^12^ or an equal volume of saline injection in the control group. Mortality in the takotsubo model was 2%. For all procedures, animals were induced with 5% isoflurane in oxygen and anaesthesia was maintained with 2% isoflurane throughout. For euthanasia, rats were anaesthetized with 5% isoflurane and injected with heparin (250 IU) and carprofen (0.33 mg).

### 2.3 Study protocol

All *in vivo* imaging or *ex vivo* isolated mitochondrial/cardiomyocyte work investigations were performed 72 h (Day 3) or 7 days (Day 7) after isoproterenol or vehicle injection. In addition, a separate group of animals was sacrificed at the same time points to provide LV tissue (divided into apex, mid-cavity, and base), snap-frozen, and later subjected to protein, gene expression, and metabolite analysis.

### 2.4 Micro PET/CT in vivo imaging

^18^F-FDG cardiac PET scanning of control and takotsubo rats (*n* = 8 per group, Day 3) was performed after a 4-h fast followed by intravenous administration of ^18^F-FDG (4.39–8.13 MBq/100 g) and 60-min list-mode PET acquisition (250–700 keV energy window) on SEDECAL SuperArgus PET/CT scanner (SEDECAL, Madrid, Spain).^14^

### 2.5 Real-time quantitative PCR

Control and takotsubo LVs (*n* = 10 each group, Day 3) were homogenized and total RNA isolated using miRNeasy columns (Qiagen). Primers are shown in Supplementary material online, *Table S1*.

### 2.6 Western blot analyses

Proteins were extracted from apical LV samples (*n* = 12 each group, Day 3) and separated by SDS-PAGE using NuPAGE 4–12% Bis–Tris midi gels (Invitrogen) in criterion cells (Bio Rad) with MOPS SDS running buffer and transferred to nitrocellulose membranes (Biorad) using criterion blotter (Biorad). Membranes were blocked and probed for proteins of interest.

### 2.7 Metabolomics

Control and takotsubo LV (*n* = 9 each group, Day 3) tissue was analysed by Metabolon Inc. (Durham, NC) for untargeted metabolite analysis as previously described.^15–17^

### 2.8 Metabolic activity assays

Control and takotsubo LV tissue (*n* = 8 each group, Day 3) was analysed according to manufacturers’ instructions: hexokinase activity assay (Abcam, ab136957), pyruvate dehydrogenase enzyme activity microplate assay kit (Abcam, ab109902), and malonyl-CoA ELISA (MyBioSource, MBS701511).

### 2.9 Cardiomyocyte isolation

Cardiomyocytes were isolated on Day 3 (*n* = 7 each group) and Day 7 (*n* = 7 each group) using a method previously defined^18^ (Supplementary material online, *methods*).

### 2.10 Sarcomere shortening and fluorescence measurements in field-stimulated cardiomyocytes

Sarcomere length was measured together with either NAD(P)H (reduced) and FAD (oxidized) (autofluorescence, *n* = 24/3 takotsubo, *n* = 25/3 control Day 3 and *n* = 29/3 takotsubo, *n* = 23/3 control Day 7) or [Ca^2+^]_c_ (indo-1 AM, *n* = 21/2 per group Day 3 and *n* = 20/2 per group Day 7) using a customized Ionoptix setup (Ionoptix, Massachusetts, USA) as previously described.^19^ Cardiomyocytes were field stimulated at 0.5 Hz before submitted to a physiological stress protocol [5 Hz stimulation and β-adrenergic challenge with isoprenaline (30 nM)]. Recovery period was initiated washing out isoprenaline and returning stimulation to 0.5 Hz.

### 2.11 Measurements of [Ca^2+^]_m_ and [Ca^2+^]_c_ in patch-clamped cardiomyocytes

Cytosolic and mitochondrial [Ca^2+^] (rhod-2, Indo-1) were measured (*n* = 18/4 takotsubo, *n* = 19/4 control Day 3 and *n* = 21/5 takotsubo, *n* = 22/5 control Day 7) using a patch-clamp-based approach employing a stress protocol previously described.^19^

### 2.12 Isolated mitochondria

#### 2.12.1 Seahorse XF and Clark electrode

Mitochondria were isolated from the myocardium of control and takostubo rats (*n* = 7 per group, Days 3 and 7) as described in Supplementary material online, methods. Mitochondrial oxygen consumption rate (OCR) was measured at 37°C using an XF24 analyser (Seahorse Bioscience, Agilent Technologies) as previously described^20,^^21^ and with a Clark oxygen electrode (Hansatech)^22^ (Supplementary material online, *methods*).

#### 2.12.2 H_2_O_2_ emission

H_2_O_2_ emission from mitochondria (*n* = 7 per group, each for Days 3 and 7) was measured using Amplex^®^UltraRed (AUR, 50 µM) (Life Technologies, Molecular Probes^®^) in a method previously described^22^ (Supplementary material online, *methods*).

### 2.13 Statistical analysis

Analysis was performed with GraphPad Prism (GraphPad Software, La Jolla, CA, USA). All data are presented as mean ± SEM. Comparison between groups was performed using unpaired two-tailed *t*-test, one-way or two-way analysis of variance (ANOVA) as appropriate, with *post hoc* Bonferroni corrections for multiple comparisons. Significance was set at *P* < 0.05.

## 3. Results

The *Graphical Abstract* summarizes metabolic abnormalities detected at day 3; proteins, metabolites and regulators which are upregulated/increased (red) and decreased/downregulated (blue) in takotsubo vs control.

These are presented further herewith together with additional findings at day 3 and 7.

### 3.1 Increased ^18^F-FDG PET uptake in the takotsubo rat heart

**Figure 1:**
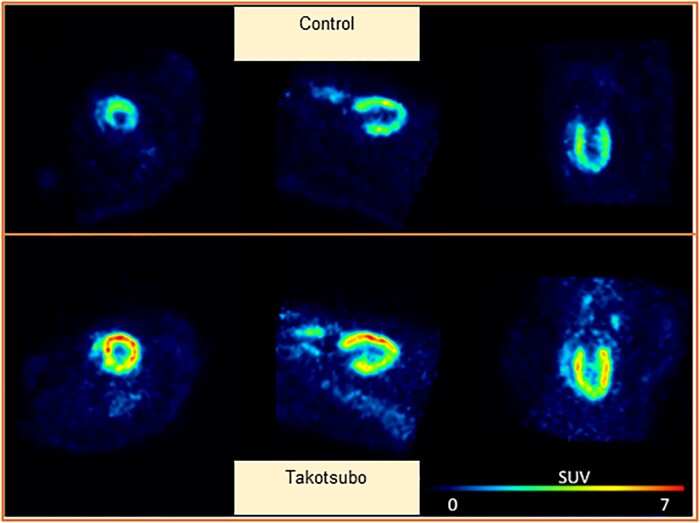
Heat map images of standardized uptake value (SUV) of ^18^F-FDG at the end of the 60-min scan for control vs. takotsubo rat shown in short-, horizonal long-, and vertical long-axis views (*n* = 8 per group, unpaired two-tailed *t*-test).

Quantitative Patlak modelling showed increased glucose metabolic rate (MRglu) in the LV of takotsubo vs. control hearts (111.9 ± 8.1 vs. 75.5 ± 6.7 mL/min/100 g, *P* = 0.006), whereas final accumulation of ^18^F-FDG (mean SUV) demonstrated a trend towards increased glucose uptake in takotsubo vs. control hearts (5.9 ± 0.7 vs. 4.2 ± 0.4, *P* = 0.06) (*Figure 1*).

### 3.2 Upregulation of glucose uptake via glucose transporters but decreased final glycolysis metabolites in the takotsubo LV

**Figure 2:**
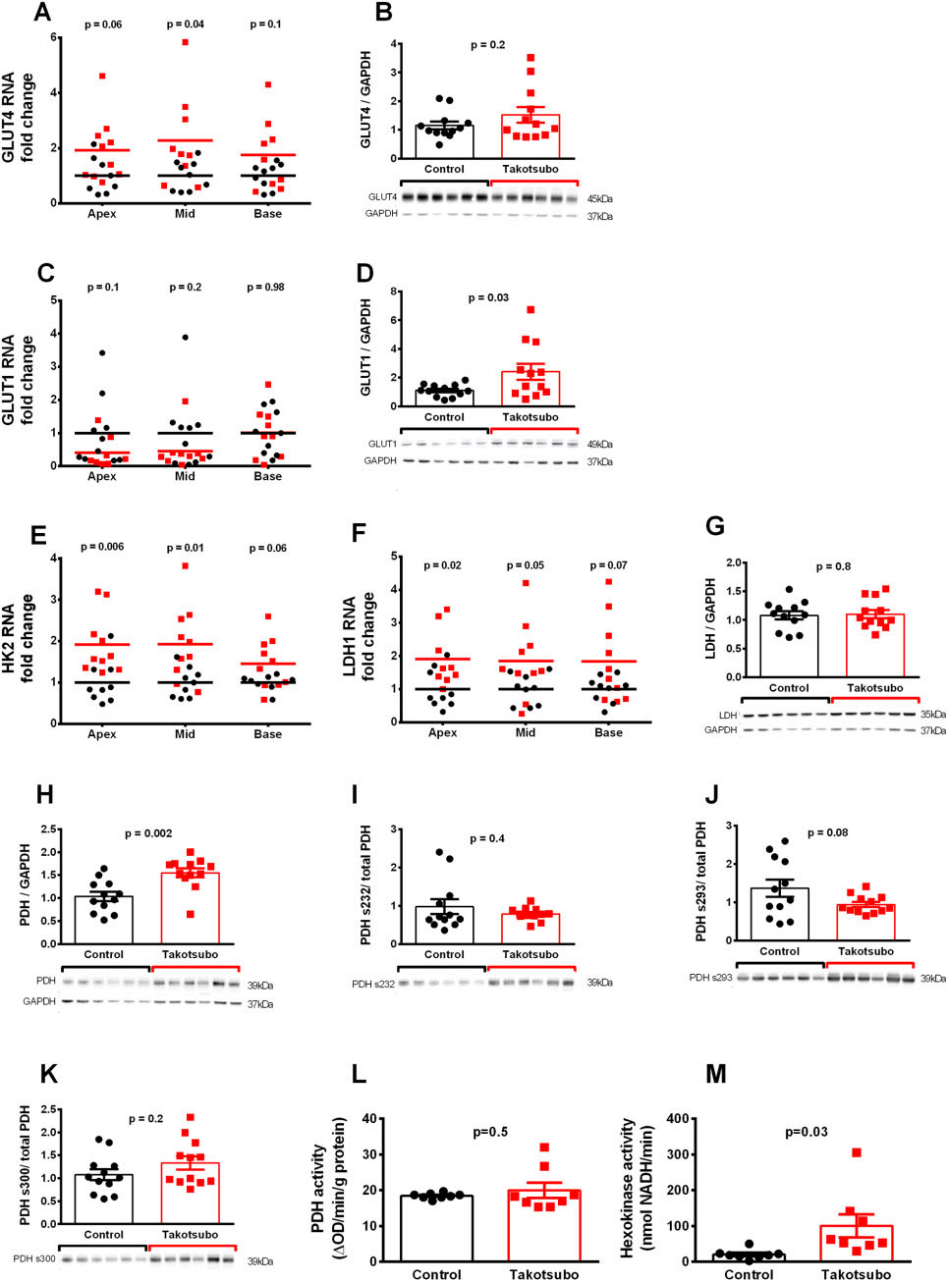
Level of RNA (*n* = 8–10 per group) protein (*n* = 12 per group) of the glycolytic pathway (*A*–*K*) and enzymatic activity (*n* = 8 each group) of PDH (*L*) and hexokinase (*M*) in control (■) and takotsubo (■) LV tissue on Day 3 with exemplary western blot analyses (unpaired two-tailed *t*-test).

GLUT4-RNA was two-fold increased in takotsubo LV relative to control, reaching statistical significance in mid-cavity (*P* = 0.04) but not in the apex or base (*P* = 0.06 and 0.1 respectively, *Figure 2A*), while GLUT4 protein levels were unchanged (1.5 ± 0.3 vs. 1.2 ± 0.1, *P* = 0.2, *Figure 2B*). GLUT1 mRNA levels were unchanged in all segments (*Figure 2C*), while GLUT1 protein was increased in the LV apex of takotsubo (2.4 ± 0.6 vs. 1.1 ± 0.1, *P* = 0.03, *Figure 2D*). Furthermore, hexokinase 2 (HK2) mRNA was two-fold increased in takotsubo LV apex and mid-cavity (*P* = 0.006 and 0.01 respectively), with a trend towards upregulation in the base (*P* = 0.06) (*Figure 2E*). In agreement with protein upregulation, enzymatic activity of HK2 was increased in the takotsubo LV (100.7 ± 32.2 vs. 20.2 ± 4.8, *P* = 0.03) (*Figure 2M*).

Metabolomic analyses revealed increased glucose-6-phosphate (1.0 ± 0.04 vs. 0.5 ± 0.07, *P* < 0.0001) and fructose-6-phosphate (1.0 ± 0.05 vs. 0.6 ± 0.07, *P* = 0.0001) in takotsubo vs. control hearts. Metabolites representing alternate fates of glucose were increased in takotsubo LV compared to control, such as UDP glucuronate (0.7 ± 0.1 vs. 0.2 ± 0, *P* = 0.005), *n*-acetylglucosamine 1-phosphate (1.2 ± 0.1 vs. 0.6 ± 0.05, *P* = 0.0003), and *n*-acetylglucosamine 6-phosphate (1.1 ± 0.7 vs. 0.8 ± 0.07, *P* = 0.02).

Lactate dehydrogenase-1 (LDH-1) transcript levels were two-fold increased in all regions, reaching statistical significance in the apex (*Figure 2F*), although apical LDH protein expression was not different (1.1 ± 0.07, *P* = 0.8, *Figure 2G*). Cardiac lactate levels were lower in takotsubo vs. control (0.9 ± 0.03 vs. 1.1 ± 0.03, *P* = 0.0005).

Total pyruvate dehydrogenase (PDH-E1α) protein levels were increased in LV apex of takotsubo vs. controls (1.6 ± 0.1 vs. 1.0 ± 0.1, *P* = 0.002, *Figure 2H*), whereas pyruvate levels were lower (0.9 ± 0.07 vs. 1.1 ± 0.04, *P* = 0.02). mRNA expression of pyruvate dehydrogenase kinase 4 (PDK4) did not differ in takotsubo vs. controls hearts (apex, 1.4 ± 0.3 vs. 1.0 ± 0.2, *P* = 0.3; mid-cavity, 1.6 ± 0.3 vs. 1.0 ± 0.1, *P* = 0.07; or base, 1.3 ± 0.2 vs. 1 ± 0.1, *P* = 0.2). Likewise, no differences between takotsubo and control hearts occurred in the proportion of PDH phosphorylated at each of the phosphorylation sites (serine 232 (0.8 ± 0.05 vs. 1 ± 0.2, *P* = 0.4), serine 293 (0.9 ± 0.07 vs. 1.4 ± 0.2, *P* = 0.08), serine 300 (1.3 ± 0.1 vs. 1.1 ± 0.1, *P* = 0.2, *Figure 2I, J, and K*), or in the enzymatic activity of PDH (20.0 ± 2.1 vs. 18.4 ± 0.3, *P* = 0.5, *Figure 2L*).

### 3.3 Fatty acid availability and their acyl-carnitine metabolites decreased in the takotsubo LV on Day 3

**Figure 3:**
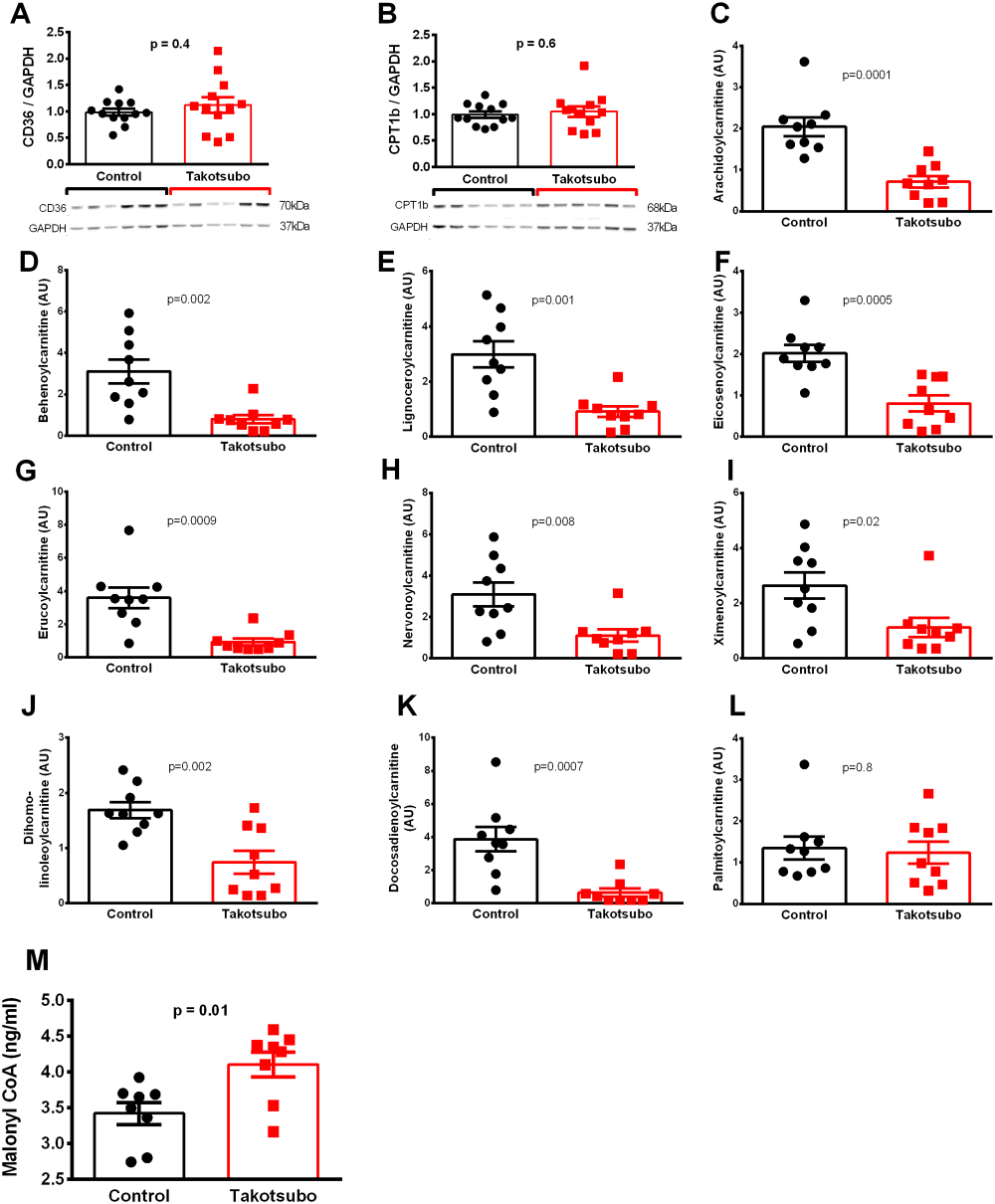
Levels of protein (*A* and *B*) and metabolites (*C*-*M*) of the fatty acid metabolic pathways in LV tissue of control (■) and takotsubo (■) rats on Day 3 (*n* = 12 for protein, *n* = 9 for metabolomics in each group, *n* = 8 malonyl-CoA unpaired two-tailed *t*-test).

The mRNA levels of fatty acid uptake protein CD36 were not significantly different in the apex (1.2 ± 0.1 vs. 1 ± 0.2, *P* = 0.3), mid (1.3 ± 0.2 vs. 1.0 ± 0.1, *P* = 0.2), or base (both 1 ± 0.2, *P* = 0.9). Likewise, protein levels of CD36 were unchanged in takotsubo vs. control LV apex (1.1 ± 0.1 vs. 0.98 ± 0.07, *P* = 0.4, *Figure 3A*).

Metabolomic analyses of the LV revealed decreased concentrations of long-chain fatty acids in takotsubo hearts vs. controls (arachidate, 1.0 ± 0.07 vs. 1.2 ± 0.04, *P* = 0.01; myristate, 1.0 ± 0.04 vs. 1.1 ± 0.02, *P* = 0.03; nonadecanoate, 0.9 ± 0.1 vs. 1.2 ± 0.04, *P* = 0.05; and stearate 0.9 ± 0.05 vs. 1.1 ± 0.02, *P* = 0.03), while palmitate levels were unchanged (0.98 ± 0.03 vs. 1.0 ± 0.001, *P* = 0.1).

Despite a six-fold increase in Carnitine Palmitoyltransferase-1b (CPT1b) mRNA in the apex (*P* = 0.03) and mid (*P* = 0.008) and a three-fold increase in the base (*P* = 0.002) of the takotsubo rat LV, there was no significant change in CPT1b protein quantity in the apex (*Figure 3B)*. Further downstream, the levels of most long-chain acyl-carnitines were decreased in the takotsubo LV (*Figure 3C–K*), with the sole exception of palmitoyl-carnitine, which was unchanged (*Figure 3L*). Long-chain acyl CoA dehydrogenase (LCAD) mRNA levels did not differ significantly from control in the apex (1.5 ± 0.2 vs. 1.0 ± 0.2, *P* = 0.1), mid (1.6 ± 0.3 vs. 1.0 ± 0.2, *P* = 0.1), or base (1.3 ± 0.3 vs. 1.0 ± 0.2, *P* = 0.4). Transcript levels of the gene encoding 3-ketoacyl-CoA thiolase (3KAT) were increased in the mid-cavity of the takotsubo LV relative to control (2.3 ± 0.4 vs. 1.0 ± 0.1, *P* = 0.004), while the increases in the apex (1.9 ± 0.4 vs. 1.0 ± 0.2, *P* = 0.05) and base (1.8 ± 0.4 vs. 1.0 ± 0.1, *P* = 0.1) were not significant. The level of malonyl-CoA, which inhibits CPT1, was increased in the takotsubo LV compared to control (4.1 ± 0.2 vs. 3.4 ± 0.2, *P* = 0.01, *Figure 3M*).

### 3.4 Decreased Krebs cycle intermediates and ATP in the takotsubo LV on Day 3

Several Krebs cycle intermediates were reduced in the takotsubo LV vs. control: α-ketoglutarate (1.1 ± 0.09 vs. 1.4 ± 0.1, *P* = 0.01) and succinyl-carnitine (1.1 ± 0.06 vs. 1.5 ± 0.1, *P* = 0.02). This was accompanied by a significant decrease in the concentration of the cellular ATP reporter dihydroorotate (0.8 ± 0.1 vs. 1.8 ± 0.3, *P* = 0.003).

### 3.5 No change in the expression and activation of 5′ AMP-activated protein kinase in the takotsubo apex on Day 3

The level of total AMP-activated protein kinase (AMPK) was similar in takotsubo and control LV apex (1.1 ± 0.07 vs. 1.0 ± 0.06, *P* = 0.7, Supplementary material online, *Figure S1A*) as was the proportion of phosphorylated and therefore activated AMPK (0.9 ± 0.06 vs. 1.0 ± 0.08, *P* = 0.3, Supplementary material online, *Figure S2B*).

### 3.6 Global inflammation in the takotsubo rat heart on Day 3

**Figure 4:**
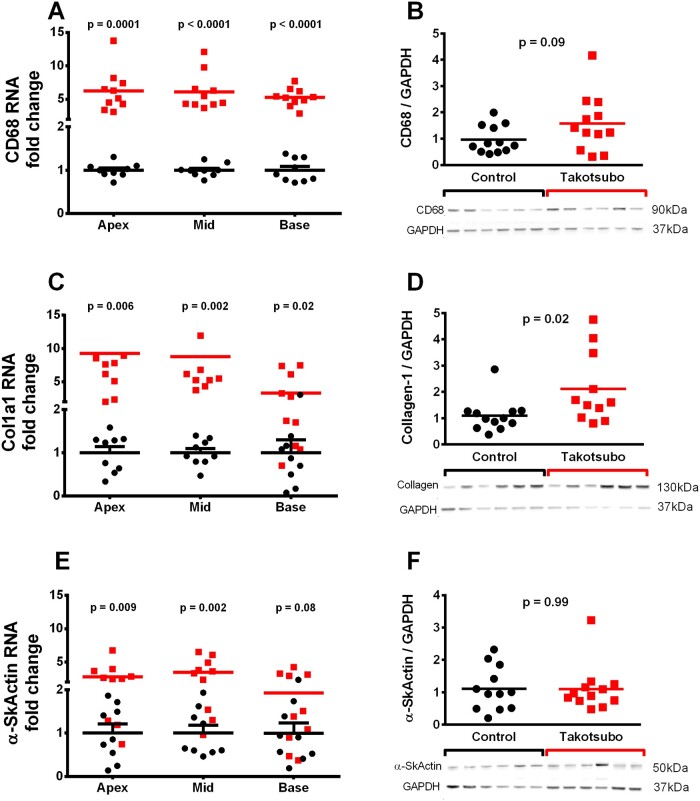
Levels of markers macrophages (CD68, *A* and *B*), fibrosis (collagen, *C* and *D*), and hypertrophy (alpha-skeletal muscle actin, *E* and *F*) in the LV of control (■) and takotsubo (■) rats on Day 3 and exemplary western blots (*n* = 11–12 for protein, *n* = 9–10 for RNA each group, unpaired two-tailed *t*-test).

There were significant increases in CD68 RNA levels in the takotsubo LV, with a six-fold increase in transcript levels relative to control in all regions (*P* < 0.0001, *Figure 4A*), while there was only a trend towards increased CD68 protein in the takotsubo apex (1.6 ± 0.3 vs. 1.0 ± 0.2, *P* = 0.09, *Figure 4B*).

### 3.7 Cardiac remodelling processes are upregulated in the takotsubo rat heart on Day 3

Transcript levels of Col1a1, which encodes the collagen type 1 alpha 1 chain were increased in all regions of the takotsubo rat LV compared to control, with a nine-fold change in the previously akinetic apex (*P* = 0.006) and mid-cavity (*P* = 0.002) and only a three-fold increase in the base (*P* = 0.02, *Figure 4C*). The protein level of collagen-1 relative to GAPDH was likewise increased in the takotsubo apex vs. control (2.7 ± 0.7 vs. 1.1 ± 0.2, *P* = 0.03, *Figure 4D*).

Alpha skeletal muscle actin (aSKA) transcription was increased two-fold in the takotsubo apex compared to controls (*P* = 0.009) and increased four-fold in mid-cavity (*P* = 0.002, *Figure 4E*). The differences in aSKA mRNA levels in the base were not significant (*P* = 0.08). The protein level of aSKA was the same in the LV apex of takotsubo rats and control (both 1.1 ± 0.2, *P* = 0.99, *Figure 4F*).

### 3.8 Isolated mitochondria from the takotsubo rat LV maintain functional integrity

**Figure 5:**
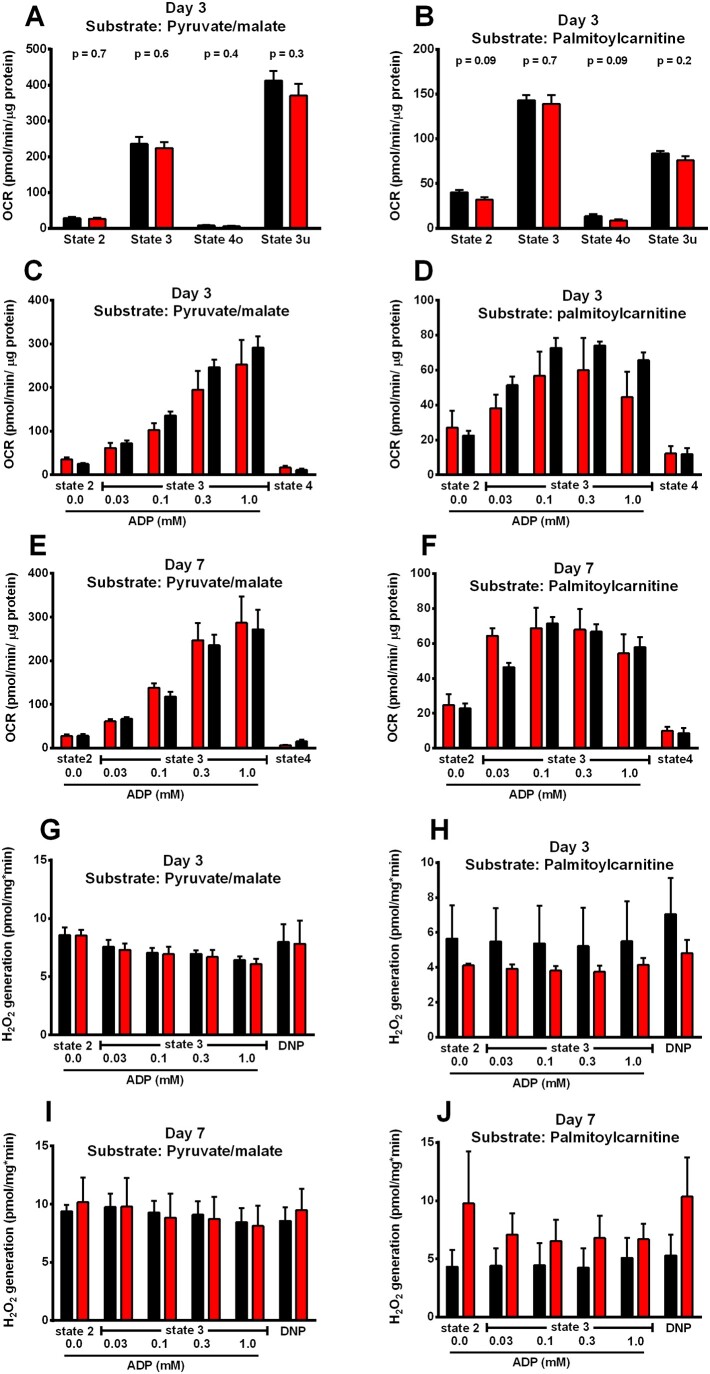
Mitochondrial OCR and ROS generation in control (■) and takotsubo (■)-isolated cardiac mitochondria, measured by Seahorse XF (*A* and *B*) or Clark electrode (*C*–*F*). H_2_O_2_ generation determined by Amplex red assay (*G*–*J*) (*n* = 7 per group; ANOVA with Bonferroni correction).

To understand whether transient LV dysfunction is related to mitochondrial defects, we analysed respiration and hydrogen peroxide (H_2_O_2_) emission in isolated cardiac mitochondria from takotsubo and control hearts. Mitochondrial OCR was measured by seahorse XF analysis or Clark electrode in the presence of either pyruvate-malate (resembling glucose-coupled fuel provision) or palmitoyl-carnitine, representing fatty acid-mediated acetyl-CoA supply. Using either substrates, respiration in the absence (State 2) or presence of increasing concentrations of ADP (State 3) and after blockade of the ATPase with oligomycin (State 4 respiration) was unchanged at Days 3 and 7, respectively (*Figure 5A–F* and Supplementary material online, *Tables S2–S4*). Similarly, the emission of H_2_O_2_, determined by Amplex Red assay, was unchanged at States 2 and 3 respiration as well as after mitochondrial uncoupling with the protonophore 2,4-dinitrophenol at Days 3 and 7 and with either substrate, respectively (*Figure 5G–J* and Supplementary material online, *Tables S5* and *S6*).

To match energy supply with demand, mitochondria take up Ca^2+^ during elevations of workload to stimulate key dehydrogenases of the Krebs cycle.^23^ However, excessive uptake of Ca^2+^, such as during situations of cytosolic Ca^2+^ overload, can trigger the opening of the permeability transition pore (PTP), inducing apoptotic or necrotic cell death.^23^ Therefore, we determined mitochondrial Ca^2+^ uptake capacity in isolated mitochondria. While in the presence of the PTP-inhibitor cyclosporine A, the rate of uptake of Ca^2+^ into mitochondria and mitochondrial buffering capacity did not differ between groups, there was a trend towards earlier opening of the PTP in the absence of cyclosporine A in takotsubo vs. control mitochondria at Day 3, which vanished at Day 7 (Supplementary material online, *Figure S2*).

### 3.9 Less efficient mitochondrial Ca^2+^ uptake oxidizes pyridine nucleotides during workload transitions in takotsubo cardiomyocytes

**Figure 6:**
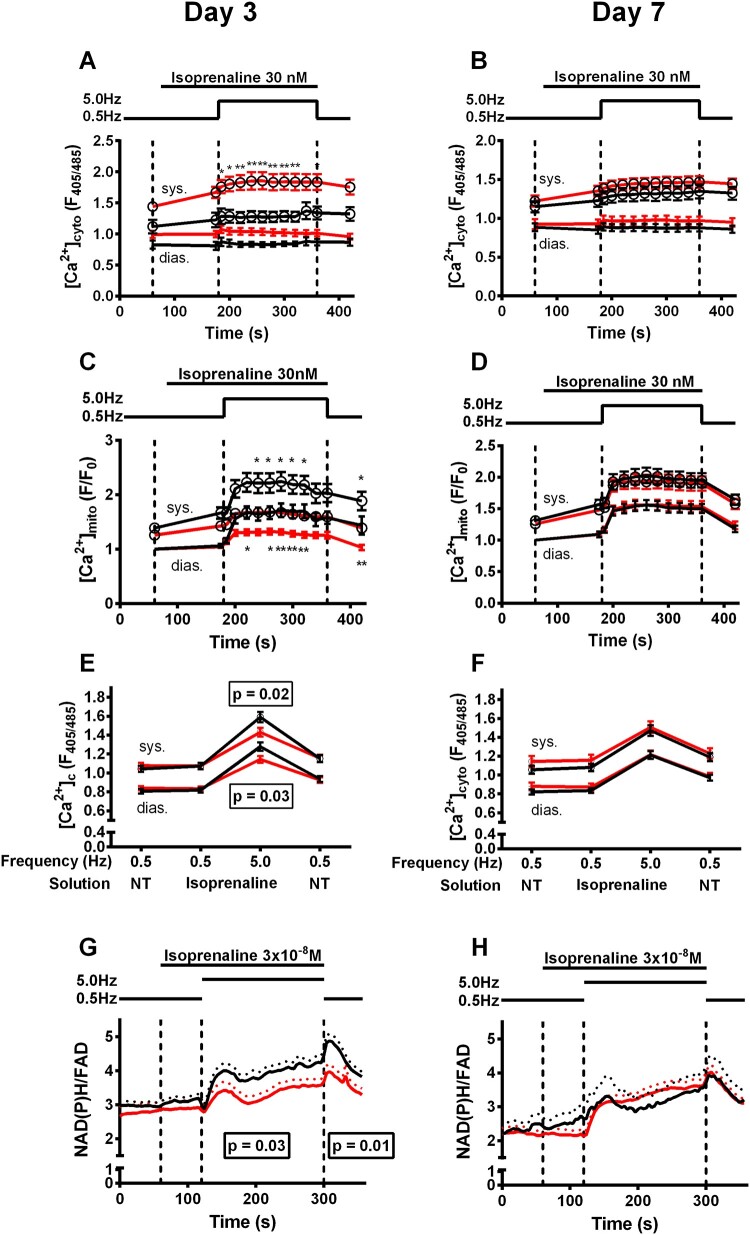
and in ;Cytosolic calcium [Ca^2+^]_c_ (*A* and *B*) and mitochondrial calcium [Ca^2+^]_m_ (*C* and *D*) in control (■) and takotsubo (■) cardiomyocytes. [Ca^2+^]_c_ fluorescence (*E* and *F*) NAD(P)H/FAD autofluorescence (*G* and *H*) in cardiomyocytes. *P < 0.05, **P < 0.01 (*n* = 18/4 takotsubo, *n* = 19/4 control Day 3 and *n* = 21/5 takotsubo, *n* = 22/5 control Day 7; ANOVA with Bonferroni correction).

In cardiac myocytes, mitochondrial function is under the control of ADP and Ca^2+^, both of which are channelled to mitochondria *via* microdomains that may be lost when mitochondria are analysed in isolation. Therefore, we wished to determine energy supply-and-demand matching in isolated and beating cardiac myocytes with mitochondria embedded into their physiological environment, in which an increase in workload is simulated by the combined application of β-adrenergic stimulation (with isoproterenol) and increased stimulation rate (from 0.5 to 5 Hz). To this end, we employed a patch-clamp based approach we previously established,^24^ in which cytosolic [Ca^2+^]_c_ is monitored together with mitochondrial Ca^2+^ concentrations ([Ca^2+^]_m_), using two different Ca^2+^ indicators (Indo-1 and rhod-2) in the same cells, respectively. In these experiments, the cytosol is equilibrated with a patch-clamp solution that contains defined concentrations of ions and Mg-ATP. Under these conditions, both diastolic and systolic [Ca^2+^]_c_ were higher in takotsubo vs. control cardiac myocytes on Day 3 (*Figure 6A*), while [Ca^2+^]_m_ transients and accumulation were reduced (*Figure 6C*). These differences resolved completely by Day 7 (*Figure 6B and D)*.

**Figure 7:**
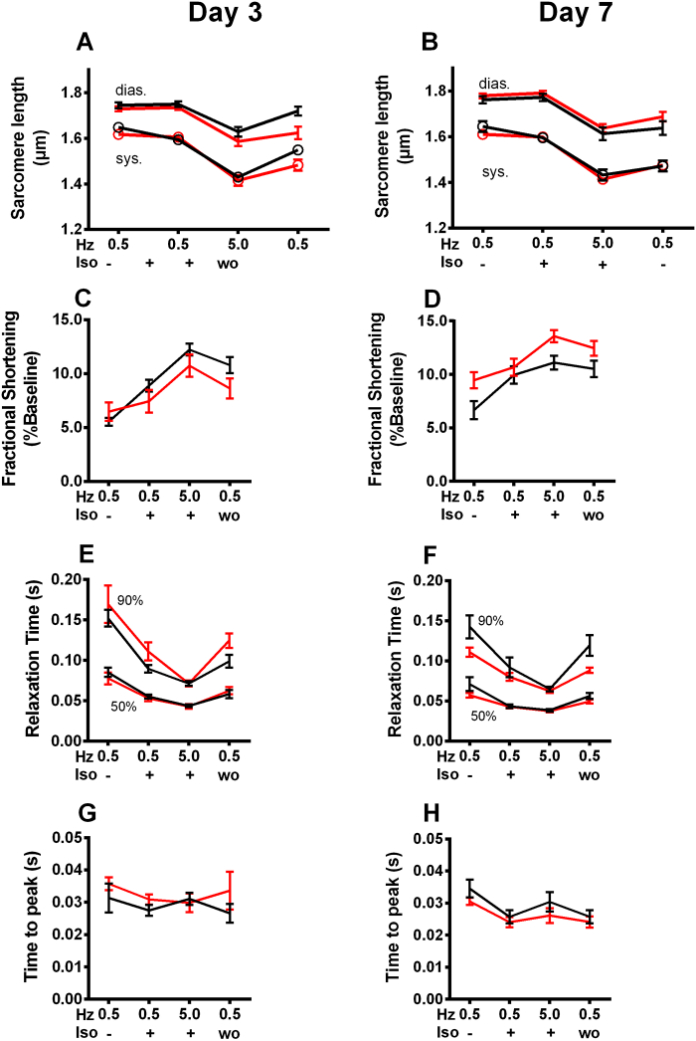
Contractile kinetics measured throughout stress protocol in cardiomyocytes isolated from control (■) and takotsubo (■) cardiomyocytes. There was no change in sarcomere length during systole or diastole at Day 3 (*A*) or 7 (*B*). Similarly fraction fractional shortening as a percentage of baseline on Day 3 (*C*) and 7 (*D*) was unchanged in takotsubo cells. The time taken to reach 50% and 90% of total relaxation did not differ at 3 (*E*) or 7 days (*F*) and the time to peak contraction was the same in control and takotsubo cardiomyocytes at 3 (*G*) and 7 (*H*) days (*n* = 7 each group at Day 3 and *n* = 7 each group at Day 7; ANOVA with Bonferroni correction).

To determine whether impaired mitochondrial Ca^2+^ uptake in cardiac myocytes of takotsubo hearts induces an energetic mismatch, we determined the redox states of NAD(P)H and FAD in intact (non-patched) cardiac myocytes exposed to a similar protocol with a workload transition induced by 5 Hz and β-adrenergic stimulation. An important difference compared to the patch-clamp conditions is that here, the intracellular milieu is unaffected by the contents of a defined pipette solution (e.g. sodium concentration is clamp on 12 mM). After isoproterenol injection, the dynamic regulation of diastolic and systolic sarcomere length as well as the kinetics of sarcomere shortening and re-lengthening in response to the workload transition were unchanged between takotsubo and control myocytes (*Figure 7A, C, E, and G*), despite a modest decrease in systolic and diastolic [Ca^2+^]_c_ in takotsubo myocytes at 5 Hz/isoproterenol (*Figure 6E*) on Day 3.

The redox state of NAD(P)H and FAD, as determined by their autofluorescence, respectively, was at the beginning of the protocol similar between both conditions (*Figure 6G*). In response to the abrupt workload transition, an initial and brief oxidation of the NAD(P)H/FAD redox state was observed (‘undershoot’) in control myocytes, followed by a substantial reduction of the redox state during the 5 Hz stimulation. From previous studies from our own lab^19^ as well as from seminal experiments by Brandes and Bers,^25^ it is well known that the reduction of the NADH redox state during such workload transitions is mediated by Ca^2+^ entering mitochondria and stimulating the Krebs cycle.^23,^^26^ On the other hand, at the end of the 5 Hz stimulation, the overshoot of the NAD(P)H/FAD redox state is explained by maintained high Krebs cycle turnover rate, which ceases when Ca^2+^ leaves the mitochondria again at low stimulation rates.^26^ In fact, and in line with the compromised mitochondrial Ca^2+^ uptake in takotsubo cardiac myocytes, the reduction of the NAD(P)H/FAD redox state during the workload transition was substantially blunted in takotsubo vs. control cardiac myocytes (*Figure 6G*), indicative of a mismatch of energy supply and demand. All defects completely resolved on Day 7 (*Figure 6B, D, F, and H*).

### 3.10 Markers of oxidative stress unchanged in takotsubo apical LV on Day 3

Since in principle, oxidation of the mitochondrial redox state may provoke increased emission of reactive oxygen species (ROS),^19^ we determined the markers of oxidative stress and the expression of antioxidative enzymes in takotsubo and control hearts. However, protein levels of peroxiredoxins (Prx) were unchanged in the takotsubo apex vs. control: Prx I (both 0.3, *P* = 0.8), Prx II (0.8 vs. 0.9, *P* = 0.6), Prx III (both 1.8, *P* = 0.99), and Prx-SO3 (0.4 vs. 0.3, *P* = 0.8). Likewise, catalase (1.3 vs. 1.2, *P* = 0.9) and haem oxygenase 1 (2.9 vs. 1.6, *P* = 0.2) expressions were not significantly altered.

### 3.11 Alternate metabolites present in the takotsubo heart tissue

Sucrose was significantly lower in the LV of takotsubo rats compared to control (2.6 ± 1.0 vs. 36.6 ± 7.5, *P* = 0.0001). Likewise, mannitol/sorbitol was significantly lower in takotsubo compared to control (0.5 ± 0.03 vs. 5.0 ± 0.8, *P* < 0.0001). The level of NAD was also lower in the takotsubo heart vs. control (1.0 ± 0.1 vs. 1.5 ± 0.1, *P* = 0.01). These sugar alcohols can result either from glucose/fructose in an NADPH-dependent mechanism or from direct esterification of fatty acids.

## 4. Discussion

The main findings of this study were: (i) There was altered substrate uptake and metabolism in the takotsubo rat heart, specifically: an increase in myocardial glucose uptake was followed by accumulation of early glycolysis sugar phosphates as well as significant increase in metabolites representing alternative terminal fates of glucose. However, despite a uniform increase of gene/protein expression of the glycolytic pathway, there was reduced availability of final glycolysis metabolites, lactate and pyruvate. In contrast, the fatty acid pathway exhibited an overall reduction in both cytoplasmic substrate availability as well as downstream mitochondrial beta-oxidative metabolites. Gene upregulation of key mitochondrial fatty acid transporters and regulators of beta-oxidation was not matched by protein upregulation. Malonyl-CoA, the key regulator of CPT-1 that mediates fatty acid entry and oxidation in mitochondria, was upregulated. The final outcome was decreased Krebs cycle intermediates and ATP generation. (ii) This was accompanied by a mild energetic deficit and aberrant Ca^2+^ handling in isolated cardiomyocytes despite functional integrity of the isolated mitochondria and preserved contractile/lusitropic properties of cardiomyocytes either at rest or during inotropic stimulation. (iii) This early stage of disease was also characterized by a global inflammatory response and upregulation of cardiac remodelling processes.

How to reconcile such multiple changes observed at almost all levels of the main metabolic pathways of the cardiac myocyte? There are four main possibilities that can be considered for discussion: (i) an overall increased metabolism which results in exhaustion of the final Krebs cycle intermediates, NAD, and ATP generation; (ii) conversely, a metabolic shut-down with low input of trans-membranous cellular substrate or early metabolic pathway diversions; (iii) an increase in final metabolites’ utilization; and (iv) a cross-regulation of glycolysis and beta-oxidation with enhancement of glycolysis and inhibition of beta-oxidation (akin to Randle adaptation).

The increased cardiac ^18^F-FDG PET uptake reported here cannot differentiate between myocyte uptake vs. metabolically active inflammatory macrophages. Accumulation of ^18^F-FDG is associated with increased macrophage activity in cardiac inflammation after acute myocardial infarction,^27^ and we have previously shown that macrophage infiltration in the takotsubo rat heart peaks on Day 3^11^ and also characterizes the human condition during the acute phase.^28^ Here, we show a substantial increase in macrophage marker CD68, which re-affirms the global inflammatory response previously seen in takotsubo.^11^ Increased glucose uptake could therefore be driven by non-myocardial cells, such as the activated macrophages, we have previously observed as well as an upregulation of the early steps of the glycolytic pathway in the myocyte. The exact mechanism would require stable isotope/flow cytometry studies, and we acknowledge that this as a limitation to our work.

Despite increased substrate availability suggested by cardiac ^18^F-FDG PET, the levels of the end product of glycolysis, i.e. pyruvate, as well as of lactate, which is produced under anaerobic conditions, were reduced in our model. It is therefore interesting that the GLUT1 rather than the GLUT4 membrane transporter was upregulated, since GLUT1 is predominantly involved in myocardial stress responses^29–31^ as well as being the predominant isoform present in infiltrating macrophages. Likewise, with the sole exception of palmitate, we observe decreased free fatty acids as well as long-chain acyl-carnitines, which is intriguing in the presence of unchanged levels of CPT1b protein levels. However, malonyl-CoA was significantly upregulated in Takotsubo hearts, suggesting at least a degree of downregulation or shift away from beta-oxidation pathways. The sole exception of palmitate is intriguing, as palmitate is by far the most abundant saturated fatty acid in the body (up to 30% of all fatty acids). Therefore, it is possible that either its abundance overcomes the dysregulations present in the beta-oxidative pathway or that its utilization remains preferentially protected until very late stages. The upregulation of thiolases (3KAT), which are directed specifically to the long-chain fatty acid oxidative pathway, may point towards the latter, i.e. a preferential utilization of specific (or most abundant) fatty acids such as palmitate.

The gene/protein upregulation of cytoplasmic/mitochondrial glycolysis and gene upregulation of fatty acid metabolic regulators in the presence of decreased metabolites can also be interpreted in one of the four scenarios presented above: (i) myocardial metabolism is unusually enhanced, functioning in ‘overdrive mode’ and so are its regulatory pathways, with the net result of metabolite exhaustion and energetic deficit or (ii) myocardial metabolism is reduced (‘shut-down’ or ‘stunned’) while the regulatory pathways attempt to compensate for decreased mitochondrial energy production and restore ATP levels. Without performing stable isotope studies to examine the turnover of each metabolic pathway, it is not possible to distinguish which possibility is more likely. While a direct relevance regarding increased consumption of final metabolite is less obvious (as third possibility), the final proposed mechanism of a cross-over regulation of the two pathways (glycolysis-beta oxidation) remains plausible. If that were to be the case, an enhanced glycolysis and consequent downregulation of beta-oxidation (*via* upregulation of malonyl-CoA, thus explaining the lack of protein upregulation of CPT-1 despite its gene upregulation) would be a similar phenomenon of competition between the two metabolic pathways described as the Randle cycle. In that case, the takotsubo heart would be a totally new metabolic phenotype, different from the classical ischaemic myocardium, where glycolytic pathways are increased as a direct result of hypoxic stress signalling^32^ or the diabetic/obese cardiomyopathy, where beta-oxidation pathways become predominant.^33^ The oxygen consumption rates appeared comparable when stimulated with either pyruvate-malate or palmitoyl-carnitine (simulating glycolytic or beta-oxidative pathways respectively) and were no different to control. The lack of increased mitochondrial respiration as well as the absence of reactive oxygen species would also favour an enhanced glycolysis and reduction of beta-oxidation. Furthermore, our work provides robust evidence that mitochondria remain intact post-takotsubo insult, which is *sine qua non* for the rapid functional recovery. The finding of intact sarcomere shortening in isolated cardiomyocytes supports sarcomeric integrity. Together, these findings point towards preserved viability and potential to regain function even in the aftermath of significant metabolic stress, leading to contractile *recovery* rather than contractile *reserve*. The unaltered AMPK signalling could similarly point to the lack of ischaemic stress signalling with preserved viability and potential for contractile recovery of the takotsubo myocyte.

This metabolic phenotype described results in the accumulation of alternate fates of glucose metabolites as shown for the first time in this report. It also explains previous findings of toxic intramyocardial lipid accumulation^12^ (degraded and thus non-metabolically useful) as well as reduced long-chain fatty acid uptake 3–5 days in post-takotsubo patients.^10^ Furthermore, our findings provide further support to the nitrosative stress hypothesis proposed by Surikow *et al.*^4,^^5^ based on the observation that the Poly(ADP-ribose) polymerase-1 (PARP-1) activation noted in their studies is poised to explain the reduction of fatty acids oxidative metabolites (*via* SIRT-1 mechanism^34^) observed in our study. This strengthens the notion that PARP-1 inhibition could be a therapeutic option for Takotsubo, in this case favourably modulating the deranged metabolism as well as ameliorating nitrosative stress.

An impaired energetic status was confirmed in our takotsubo model, as the NAD(P)H/FAD ratio was more oxidized during β-adrenergic stimulation at Day 3, while this mismatch resolved by Day 7: this is in contradistinction with the human disease where a degree of energetic impairment persists. The mismatch between energetic supply and demand was evident during increased workload, whereas energetic impairment was detectable at rest in previous human studies,^2^ and was never studied under stress circumstances. Such differences may derive from simplistic creation of the rat model compared to the complex human condition, or the presence of comorbidities in humans (which may be indirectly augmenting such a deficit), or more advanced age of human patients.

Our investigations show defective mitochondrial Ca^2+^ uptake in isolated cardiomyocytes on Day 3, but no change in the ability of isolated mitochondria to sequester Ca^2+^. We also saw an increase in the concentration of Ca^2+^ in the cytosol during systole and diastole during increased workload. This finding is in-keeping with measurements of increased Ca^2+^ concentration in iPSCs isolated from takotsubo patients.^35^

Since Ca^2+^ is a key regulator of metabolism and energetics by means of its regulation of the Krebs cycle,^36^ it may also be related to the observed energetic deficit. We have demonstrated depressed Krebs cycle activity in the takotsubo LV at the same timepoint as defective Ca^2+^ handling. Furthermore, we have previously observed that inhibition of mitochondrial Ca^2+^ uptake induces oxidation of NAD(P)H and FAD and so decreased mitochondrial Ca^2+^ uptake may be driving the energetic mismatch in takotsubo cardiomyocytes.^19,^^23,^^24^ In fact, studies on mice lacking the mitochondrial Ca^2+^ uniporter revealed that the inotropic response to β-adrenergic stimulation is compromised and delayed.^37,^^38^ Therefore, defective mitochondrial Ca^2+^ uptake may plausibly contribute to an energetic deficit that becomes limiting for cardiac function in the clinical condition of acute takotsubo cardiac dysfunction.

The exact mechanism of aberrant Ca^2+^ handling in takotsubo cardiac myocytes remains to be defined. Since uptake of Ca^2+^ by isolated mitochondria was unchanged, the reason for the differences in patch-clamped cardiomyocytes may be related to an impairment of the mitochondrial Ca^2+^ microdomain, where mitofusin 2 and potentially also other proteins govern the tethering of mitochondria to the sarcoplasmic reticulum.^39^ Further studies should address this in more detail.

Abnormal myocardial Ca^2+^ handling has been linked to development of tachyarrhythmias; indeed it is during the acute stage after takotsubo insult when patients are most likely to develop malignant arrhythmias. Understanding the mechanism of aberrant Ca^2+^ handling in takotsubo may therefore highlight drug targets which could rectify repolarization abnormalities leading to ventricular arrythmias in patients. Interestingly, ventricular arrhythmias in response to ouabain, which by an increase in cytosolic Na^+^ concentrations hampers mitochondrial Ca^2+^ uptake, could be blunted when suppressing mitochondrial Ca^2+^ extrusion,^40^ indicating that the redox mismatch resulting from insufficient mitochondrial Ca^2+^ accumulation may underlie arrhythmias also in takotsubo cardiomyopathy.

Finally, we observed increased collagen expression and hypertrophic marker α-skeletal muscle actin-RNA^41,^^42^; these suggest that additional cardiac remodelling processes are present as early as Day 3 after the acute insult before established fibrosis and inflammatory changes become apparent.^11^ Since there is an established link between cardiac inflammation and onset of fibrosis, targeting inflammation early presents a viable treatment option for takotsubo.

### 4.1 Study limitations

We did not present glycolysis metabolite data between the glucose-6-phosphate and pyruvate steps, being limited by the specific metabolomic analysis utilized, neither did we study translocation of glucose and fatty acid transporters (GLUT-4 and CD36) to the plasma membrane, which could provide further support into the metabolic dysregulations observed here.

## 5. Conclusions

Our study indicates no metabolic substrate inflexibility but significant alterations in glucose and fatty acid metabolism as well as Krebs cycle activity, which will require metabolic turnover studies for confirmation and insight into whether energetics can be rescued by current cardiometabolic modulators. Metabolic changes accompany Ca^2+^ handling defects, which may underlie life-threatening arrhythmias, with both mitochondria and sarcomere functional integrity. The increase in inflammatory markers recapitulates the human disease, is associated with early cardiac remodelling processes and remains a more likely therapeutic target.

## Supplementary material

Supplementary material is available at *Cardiovascular Research* online.

## Authors’ contributions

D.K.D., C.M., M.D., N.G., M.K., A.N., A.W., and M.V.B. contributed significantly to the conception and design of the experimental work. The acquisition and analysis of the data were carried out primarily by N.G. with additional experiments carried out by M.K., A.N., L.C., C.M., M.M., L.S., and C.H. Interpretation of the data together with drafting and revising this manuscript was carried out by D.K.D., C.M., and N.G. with input from M.V.B., M.D., M.K., A.N., and A.W. All authors give permission for the publication of this final manuscript.

## Supplementary Material

cvab081_Supplementary_Data

## Notes

Translational perspective The simultaneous dysregulation in the glycolytic and beta-oxidation pathways, which underlies the energetic deficit of the takotsubo heart, supports further testing of currently available metabolic modulators as possible candidates for successful therapy, as well as targeting the inflammatory and remodelling pathways.
