# Supplementary material for: Metabolic alterations in a rat model of takotsubo syndrome
Source: Cardiovasc Res. 2021 Mar 12;118(8):1932–46. doi: 10.1093/cvr/cvab081 (PMC9239582; doi:10.1093/cvr/cvab081)

Metabolic alterations in a rat model of Takotsubo syndrome

Supplemental Materials

Nadine Godsman, BSc[1]
Michael Kohlhaas, PhD[2]
Alexander Nickel, PhD[2]
Lesley Cheyne, BSc[1]
Marco Mingarelli, BSc[3]
Lutz Schweiger, PhD[4]
Claire Hepburn, PhD[1]
Chantal Munts, BSc[5]
Andy Welch, BSc, PhD, MIPEM[3]
Mirela Delibegovic, BSc, PhD[1]
Marc Van Bilsen, PhD[5]
Christoph Maack, MD, PhD[2]
Dana K Dawson, DM, FRCP, D.Phil, FESC[1]

[1] Aberdeen Cardiovascular and Diabetes Centre**,** University of Aberdeen**,** Aberdeen, United Kingdom

[2] Comprehensive Heart Failure Center (CHFC), Würzburg, Germany

[3] Biomedical physics, University of Aberdeen, Aberdeen, United Kingdom

[4] John Mallard Scottish P.E.T. Centre, University of Aberdeen, Aberdeen, United Kingdom

[5] School for Cardiovascular Diseases, Faculty of Health, Medicine and Life Sciences Maastricht University, Netherlands

Supplemental methods

Micro PET/CT in-vivo imaging

Control and takotsubo rats (n=8 per group, day 3) were induced with 5% isoflurane in oxygen and anaesthesia was maintained with 2% isoflurane throughout. ECG electrodes were attached on the paws and the tail vein was cannulated before the animal was placed supine on the scanner bed. CT imaging was carried out with 40 kV 140 µA beam current using 360 projections. A dose of ^{18}F-FDG (4.39 – 8.13 MBq/100 g) was administered *via* the tail cannula and flushed through with heparinised saline. List-mode PET data were acquired (using a 250 keV – 700 keV energy window) in a single bed position for a total of 60 minutes. Blood glucose was monitored every 10 minutes to ensure a steady level between 10-15 mmol/L. Venous blood samples were taken every 30 minutes for quantification of specific ^{18}F-FDG concentration in the blood of each animal.

Calibration was performed on completion of acquisition using a phantom of a similar size to a rat filled with a known concentration of ^{18}F-FDG. Three-dimensional sinograms were reduced to 2-dimensions by Fourier rebinning and reconstructed using a 2D-OSEM algorithm, including corrections for dead-time, random counts, attenuation and photon scatter using the manufacturer’s software. The list-mode data were sorted into 19 timeframes with the following number-of-frames x time (seconds): 4x10, 1x20, 2x30, 3x60, 1x300, 8x600. Image analysis was completed with Pmod Software (Pmod Technologies, CH).

PET-CT image analysis

A global calibration factor was derived from the phantom acquisition and used to convert the images to units of MBq/ml. The images were then multiplied by the weight of the animal and divided by the injected activity, so that they were in units of Standardized Uptake Value (SUV). Regions of interest (ROIs) covering the myocardium and the blood were defined as follows. For the myocardium; the centre of the heart was identified on the final frame of the PET data and a spherical ROI was defined of radius 8.5mm centred on this point. The myocardium ROI was defined as all voxels within this spherical ROI that had values greater than 50% of the maximum value. For the blood; the first four frames (40 seconds) of the dynamic image were summed to produce an “early” image. A cylindrical ROI of radius 4mm was defined over the region of the abdominal aorta in this early image. The blood ROI was defined as all voxels within this cylindrical ROI that had values greater than 80% of the maximum value. The blood and myocardium ROIs were copied onto the full dynamic sequence and the average value was used to define blood and myocardium Time Activity Curves (TACs). Patlak analysis(37) was used to quantify metabolism using the blood and myocardium TACs. Finally, the slope of the Patlak plot was scaled by the average of all the plasma glucose measurements that were taken during the scan, to give a Metabolic Rate of Glucose Utilisation (MRGlu).

Real-Time Quantitative PCR (qPCR)

Synthesis of cDNA was performed according to the manufacturer’s instructions (Iscript; Biorad) and gene expression analysed by real-time PCR using iQ SYBR-Green Supermix (Biorad) and the CFX96 Touch device (Biorad). Transcript levels were normalised for hypoxanthine-guanine phosphoribosyltransferase (HPRT) using the delta Ct method and expressed as fold-change relative to control for each region.

Western Blot

Tissue lysates were produced by manually homogenising tissue using a pre-cooled pestle and mortar before adding radio immunoprecipitation assay (RIPA) buffer (50 mM tris, 150 mM NaCl, 1% Triton X-100, 0.5% sodium deoxycholate, 0.1% SDS, cOmplete™ Protease Inhibitor Cocktail tablet (Roche), 1 mM sodium orthovanadate, 1 mM sodium fluoride). They were then snap frozen, thawed and kept on ice on an agitator for 1 hour before being cleared by centrifugation (13000 rpm, 15 minute, 4°C). The supernatant was transferred and protein concentration was determined using a BCA Protein Assay kit (ThermoFisher).

Proteins were separated by SDS-PAGE using NuPAGE 4-12% Bis-Tris midi gels (Invitrogen) in criterion cells (Bio Rad) with MOPS SDS running buffer (50 mM MOPS, 50 mM Tris base, 0.1% SDS, 1 mM EDTA, pH 7.7) and transferred to 0.45 µm nitrocellulose membranes (Biorad) in transfer buffer (25 mM Tris, 0.2 M glycine, 20% methanol) using criterion blotter (Biorad). Membranes were then blocked with 5% milk or BSA in TBST (20 mM trisaminomethane, 137 mM NaCl, 0.9 mM polysorbate 20, pH 7.5) before being probed for CD68, collagen1, ASKMActin, AMPK α1+ α2 total, AMPK α1 phospho T183+ α2 phospho T172, LDH, GLUT1, PDH-E1α, PDH phospho S232, PDH phospho S293, PDH phospho S300, CD36, CPT1b, PrxI, PrxII, PrxIII, HO-1, catalase, Prx-SO3, O-GlcNAc, GAPDH or β-actin. Membranes were washed in TBST and incubated at RT for 1 hour with HRP-conjugated goat anti-mouse or anti-rabbit at 1:5000 dilution in 2% milk/BSA. After further washing luminol-based detection was performed using ECL reagent (100 mM

Tris-HCl, 2.5 mM luminol, 0.4 mM coumaric acid, 0.02% H_2O_2). Densitometry analysis was carried out using ImageJ software (Bethesda, Maryland, USA).

Metabolomics

Metabolites were extracted using a methanol-based method, after extraction organic solvents were evaporated using a TurboVap® (Zymark). Extracted samples were divided and analysed by reverse phase (RP)/UPLC-MS/MS with positive ion mode electrospray ionization (ESI), by RP/UPLC-MS/MS with negative ion mode ESI and by HILIC/UPLC-MS/MS with negative ion mode ESI.

Analysis was carried out using the Laboratory Information Management System (LIMS), data extraction and peak-identification software and data processing tools for QC and compound identification. Raw counts were normalised and rescaled to set the median equal to 1, the scaled intensity values are presented.

Cardiomyocyte isolation

Rats were anaesthetised with 5% isoflurane and injected with heparin (250 IU) and Carprofen (0.33 mg). The heart was excised and retrograde-perfused via aortic cannula at 37°C with Ca^{2+}-free perfusion buffer for 4 minutes followed by a 4-6 minutes perfusion with the enzymatic solution. To stop digestion the ventricles were cut off and immersed in stop buffer at room temperature. Cells were dissociated and the solution containing the cardiomyocytes was transferred to a 15 ml tube for an 8 minute sedimentation after which the supernatant was transferred to another tube and centrifuged (180 g, 1 minute). Both pellets were resuspended in 10 ml of culture medium (M199, 5% FCS, 1% penicillin and streptomycin, 10 mM HEPES).

Mitochondrial Isolation

Fresh LV tissue (immediately after sacrifice) was dissected out and manually homogenised in isolation buffer (sucrose 75 mM, mannitol 225 mM, HEPES 2 mM, EGTA 1 mM, BSA 4 mg/ml, protease 1.6 mg/ml, pH 7.4) for 14 minutes using a Teflon pestle. The homogenate was centrifuged (480 g, 5 minutes) and the supernatant further centrifuged (7700 g, 10 minutes) to obtain the pellet containing intact mitochondria. The pellet was washed twice in mitochondrial suspension solution (MSS; sucrose 75 mM, mannitol 225 mM, HEPES 2 mM, pH 7.4), centrifuged (7700 g, 5 minutes), and resuspended in 150 µl of MSS.

Mitochondrial Oxygen Consumption Rate (OCR)

The function of the respiratory chain in isolated mitochondria was evaluated by measuring oxygen consumption rate in the presence of substrates of glucose and fatty acid oxidation pathways. The seahorse XF analysis provides a high throughput method which has the potential to increase the sensitivity and minimise variability since both control and takotsubo mitochondria can be assessed on the same 24-well plate. The Clark electrode on the other hand is an established and well validated technique which can measure respiration in mitochondria from only one source at a time. We employed both methods here to confirm the validity of the seahorse XF method and benchmark this model’s variability with that of the Clark electrode measurements.

Seahorse XF assay

Mitochondria were isolated from the left ventricle of control and takotsubo rats (n= 12 in each group, day 3) and protein concentration was determined by the Bradford

method. The sensor cartridge was hydrated overnight at 37°C and calibrated prior to data collection. Isolated mitochondria (5 µg/well) in mitochondrial assay solution (MAS; 70 mM sucrose, 220 mM mannitol, 5 mM $MgCl_2$, 5 mM KH_2PO_4, 2 mM HEPES, 1 mM EGTA, 0.2% fatty acid free BSA, pH 7.4) were loaded in to a 24-well plate and centrifuged (2000 g, 20 min, 4°C). MAS containing respiratory substrates pyruvate/malate (10 mM/5 mM) or palmitoyl carnitine/malate (40 µM/1 mM) was added to 12 wells each and the plate was incubated at 37°C for 8 minutes before being loaded in to the XF24 analyser. Mitochondria were supplied with either pyruvate/malate (10 mM/5 mM) or palmitoyl-carnitine/malate (40 µM/1 mM) as substrates. Basal (state 2) respiration was measured before injection of ADP (4 mM) to induce maximal state 3 respiration, state 4o was measured after oligomycin (316 nM) injection and state 3u (maximal uncoupled) after carbonyl cyanide 4-(trifluoromethoxy)phenylhydrazone (FCCP, 4 µM). Antimycin-A (4 µM) was injected at the end of the assay to inhibit mitochondrial respiration and provide a background reading.

Clark Electrode

Protein concentration was determined by the Lowry method(38). Pyruvate/malate (5 mM) or palmitoyl carnitine/malate (6.25 µM/ 3 mM) were added to the chamber containing 2 ml containing respiration buffer (137 mM KCl, 2 mM KH_2PO_4, 500 µM EGTA) and 400 µg of mitochondria to initiate basal (state 2) respiration. To induce maximal state 3 respiration ADP was added at 1-minute intervals to reach final concentrations of 0.03 mM, 0.1 mM, 0.3 mM and 1 mM. Finally, State 4o respiration was initiated by addition of oligomycin (1.2 µM).

H_2O_2 emission

Mitochondrial H_2O_2 production was measured after addition of ADP (1 mM), DNP (5 µM) and antimycin A (15 µM) in the presence of pyruvate/malate (5 mM) or palmitoyl-carnitine/malate (6.25 µM/ 3 mM). Experiments were performed using the Tecan GENios Pro Reader. All experiments were conducted in respiration buffer with 30 µg mitochondria per well and H_2O_2 was quantified from a calibration curve obtained from known H_2O_2 concentrations.

Mitochondrial Ca^{2+} uptake

A calcium green assay was performed using a plate reader with reagent injectors (infinite M200Pro, Tecan). Mitochondria (165 µg) were loaded in to each well in 200 µl buffer (120 mM KCl, 70 mM mannitol, 25 mM saccharose, 20 mM HEPES, 5 mM KH_2PO_4, 20 µM EGTA). Mitochondria were incubated for 15 minutes with with K-glutamate/malate (5 mM/2.5 mM) with or without cyclosporin A (1 µmol/L). Calcium Green-5N (1 µM) was added before the assay was performed by injecting Ca^{2+} (10 µmol/L) every 2 minutes. Extramitochondrial Ca^{2+} was determined by measuring the intensity of fluorescence emission by Calcium Green-5N upon binding to free Ca^{2+}.

Supplementary figures and tables

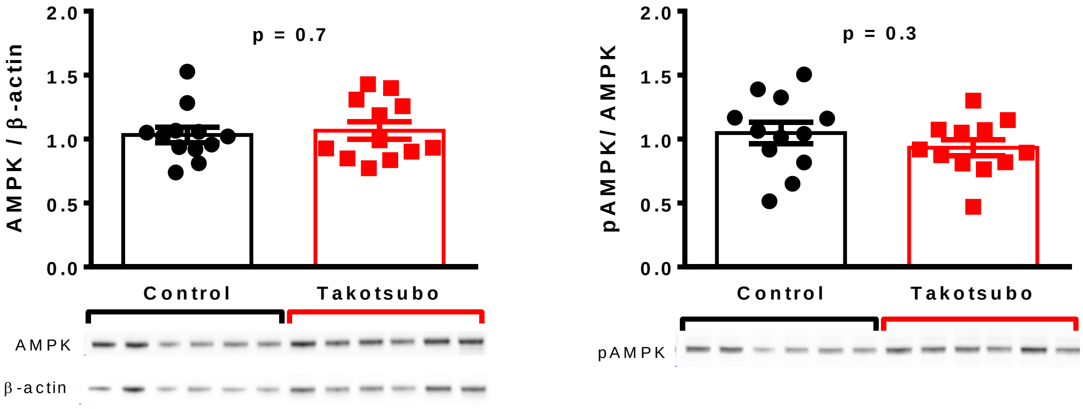

Supplemental figure 1: Expression and activation of 5' AMP-activated protein kinase (AMPK) in apical left ventricular (LV) tissue of control (■) and takotsubo-like (■) rats on day-3 and exemplary western blots.(n=12 each group, unpaired t-test)

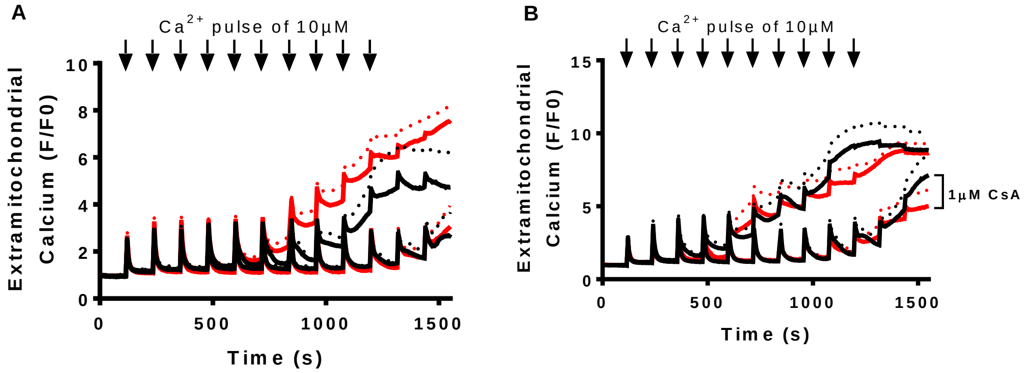

Supplemental figure 2: Changes in extramitochondrial calcium measured during calcium green assay in mitochondria isolated from control (■) and takotsubo-like (■) rats. There were no differences in calcium uptake by control and takotsubo-like mitochondria on day 3 (A) and day 7 (B) after injections of 10 µM free Ca^{2+}. (n=7 per group, at day-3 and at day-7, unpaired t-test).

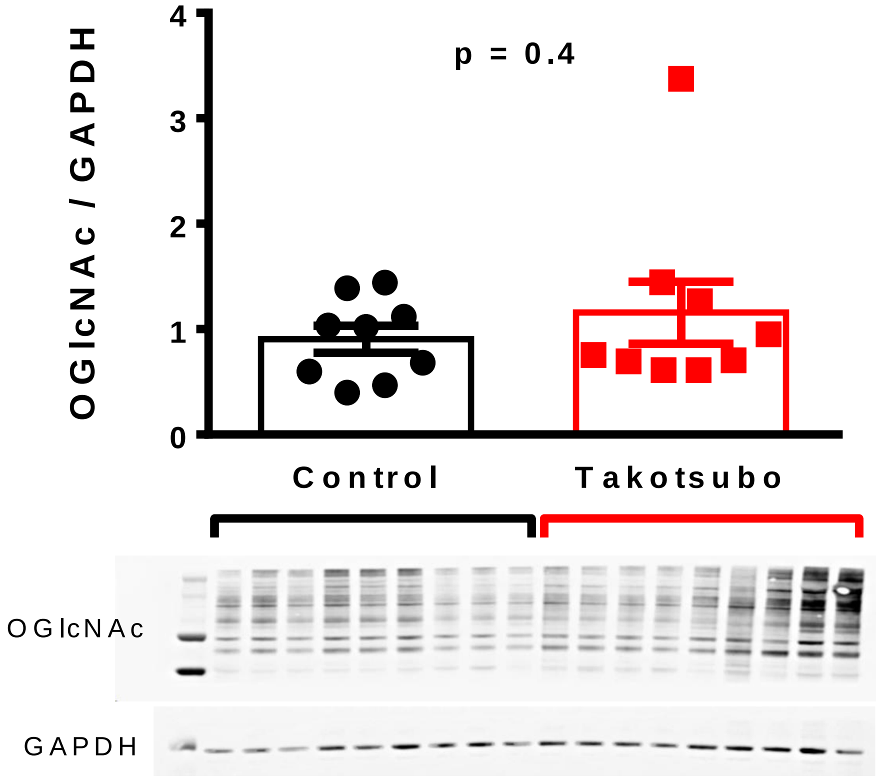

Supplemental figure 3: Levels of O-GlcNAc modified proteins in apical left ventricular (LV) tissue of control (■) and takotsubo-like (■) rats on day-3 and exemplary western blots. (n=9 per group, unpaired t-test)

Additional tables

Table 1

Transcript	Forward	Reverse
Col1a1	CGAAGGCAACAGTCGATTCA	GGTCTTGGTGGTTTTGTATTCGAT
aSKA	TGAGACCACCTACAACAGCA	CCAGAGCTGTGATCTCCTTC
CD68	ATCAACCTACCAGCCCCTCT	TGCATGCTGAAGAAATGAGG
GLUT1	CCACCACACTCACCACACTC	CCATAAGCACGGCAGACAC
GLUT4	GGAAGGAAAAGGGCTATGCTG	TGAGGAACCGTCCGAGAATGA
PDK4	GCATTTCTACTCGGATGCTCATG	CCAATGTGGCTTGGGTTTCC
HK2	TGATCGCCTGCTTATTCACGG	AACCGCCTAGAAATCTCCAGA
CD36	TTTCCTCTGACATTTGCAGGTCTA	AAAGGCGTTGGCTGGAAGAA
CPT1b	CCAGATGGAGAGGATGTTCAACA	AGAAGCGACCTTTGTGGTAGACA
LCAD	CGGCACAAAAGAACAGATCG	CTCCCAGACCTTTTGGCATT
3KAT	GGTGTCCCTGAAGTGTTGCT	TCTGTCTGCACGAATGTTCC
HPRT	GCGAAAGTGGAAAAGCCAAGT	GCCACATCAACAGGACTCTTGTAG
LDH1	AATGAAGAACCTTAGGCGGGTGC	CATCCTCATTGATTCCATAGAGACCCT

Table 1: Primer sequences for Real-time PCR target genes.

Table 2:

Respiratory State	Pyruvate			Palmitoyl Carnitine		
	Control	Takotsubo	P value	Control	Takotsubo	P value
2	28.4 ±3.6	26.5 ±3.6	0.7	40.2 ± 2.7	33.8 ±2.4	0.09
3	236.1 ±19.4	224.1 ±16.9	0.6	143.3 ±5.7	139.3 ±9.8	0.7
4o	8.1 ±1.7	5.8 ±1.8	0.4	13.4 ±2.3	8.7 ±1.4	0.09
3u	412.9 ±27.0	370.9 ±32.2	0.3	83.7 ±2.9	75.9 ±4.8	0.2

Table 2: Oxygen consumption rate (pmol/min/µg protein) of mitochondria isolated from control and takotsubo rat left ventricles on day-3 measured by seahorse XF assay. Data shown as mean±SEM.

Table 3:

Respiratory State	Pyruvate			Palmitoyl carnitine		
	Control	Takotsubo	P value	Control	Takotsubo	P value
2	35.2 ±5.1	25.3 ±1.7	0.1	27.1 ±9.6	22.4 ±2.9	0.7
3 (0.03 M ADP)	61.4 ±11.7	72.0 ±6.7	0.5	38.2 ±7.7	51.3 ±5.0	0.2
3 (0.1 M ADP)	102.8 ±42.6	136.3 ±8.4	0.1	56.8 ±13.9	72.7 ±5.7	0.3
3 (0.3 M ADP)	195.2 ±42.6	246.3 ±17.6	0.3	59.9 ±18.9	74.0 ±2.4	0.5
3 (1.0 M ADP)	252.9 ±55.7	291.5 ±25.7	0.5	44.6 ±14.5	65.7 ±4.4	0.2
4o	16.6 ±3.7	10.9 ±2.9	0.3	12.8 ±4.2	11.9 ±3.5	0.9

Table 3: Mean oxygen consumption rate (OCR) (pmol/min/µg protein) of mitochondria isolated from control and takotsubo rat ventricles on day-3 measured by Clark electrode. Data shown as mean±SEM.

Table 4:

Respiratory State	Pyruvate			Palmitoyl carnitine		
	Control	Takotsubo	P value	Control	Takotsubo	P value
2	27.5 ±3.2	28.0 ±4.1	0.9	24.7 ±6.2	22.9 ±2.8	0.8
3 (0.03 M ADP)	61.4 ±4.7	66.9 ±3.9	0.4	64.4 ±4.3	46.4 ±2.5	0.007
3 (0.1 M ADP)	138.0 ±9.9	117.9 ±11.0	0.2	68.8 ±11.6	71.4 ±3.7	0.8
3 (0.3 M ADP)	247.0 ±39.7	235.1 ±24.7	0.8	68.0 ±11.7	66.7 ±4.1	0.9
3 (1.0 M ADP)	286.9 ±60.6	271.7 ±44.7	0.8	54.32 ±54.3	57.8 ±5.8	0.8
4o	6.4 ±1.3	15.1 ±3.9	0.07	9.1 ±2.4	8.5 ±3.1	0.7

Table 4: Mean oxygen consumption rate (OCR) (pmol/min/µg protein) of mitochondria isolated from control and takotsubo rat ventricles on day-7 measured by Clark electrode. Data shown as mean±SEM.

Table 5:

Respiratory State	Pyruvate			Palmitoyl carnitine		
	Control	Takotsubo	P value	Control	Takotsubo	P value
2	8.6 ±0.7	8.6 ±0.5	1	5.6 ±1.9	4.1 ±0.1	0.4
3 (0.03 M ADP)	7.6 ±0.6	7.3 ±0.6	0.7	5.5 ±1.9	3.9 ±0.3	0.4
3 (0.1 M ADP)	7.0 ±0.4	6.9 ±0.6	0.9	5.3 ±2.2	3.8 ±0.3	0.5
3 (0.3 M ADP)	7.0 ±0.3	6.7 ±0.6	0.7	5.2 ±2.2	3.7 ± 0.4	0.5
3 (1.0 M ADP)	6.4 ±0.3	6.1 ±0.5	0.6	5.5 ±2.3	4.1 ±0.4	0.6
3u (DNP)	8.0 ±1.5	7.8 ±2.0	0.9	7.0 ±2.1	4.8 ±0.8	0.3

Table 5: Mean H_2O_2 generation (pmol/min/mg protein) in mitochondria isolated from control and takotsubo rat ventricles on Day-3 measured by amplex red assay. Data shown as mean±SEM.

Table 6:

Respiratory State	Pyruvate			Palmitoyl carnitine		
	Control	Takotsubo	P value	Control	Takotsubo	P value
2	9.4 ±0.5	10.2 ±2.1	0.7	4.3 ±1.4	9.8 ±4.5	0.3
3 (0.03 M ADP)	9.8 ±1.2	9.8 ±2.5	1	4.4 ±1.5	7.1 ±1.8	0.3
3 (0.1 M ADP)	9.3 ±1.0	8.8 ±2.1	0.9	4.5 ±1.9	6.5 ±1.8	0.5
3 (0.3 M ADP)	9.1 ±1.1	8.7 ±1.9	0.9	4.3 ±1.6	6.8 ±1.9	0.3
3 (1.0 M ADP)	8.5 ±1.2	8.1 ±1.7	0.9	5.1 ±1.7	6.7 ±1.3	0.5
3u (DNP)	8.6 ±1.2	9.5 ±1.8	0.7	5.3 ±1.8	10.3 ±3.4	0.2

Table 6: Mean H_2O_2 generation ±SEM (pmol/min/mg protein) in mitochondria isolated from control and takotsubo rat ventricles on day-7 measured by Amplex red assay. Data shown as mean±SEM.

Uncropped Western Blot Images

For all western blots 2 gels were run with n=6 for each group. The representative blots displayed in the figures in the manuscript are all the first image below for each protein. The remaining data is from the second image for each protein with the same layout of control (n=6) and takotsubo-*like* (n=6) apex. All bands were quantified as described in the methods section before being normalised to the loading control for each lane on the same gel and then normalised to the mean of the control so that data from both could be collated for a total n=12 each group.

GLUT4:

Blot 1

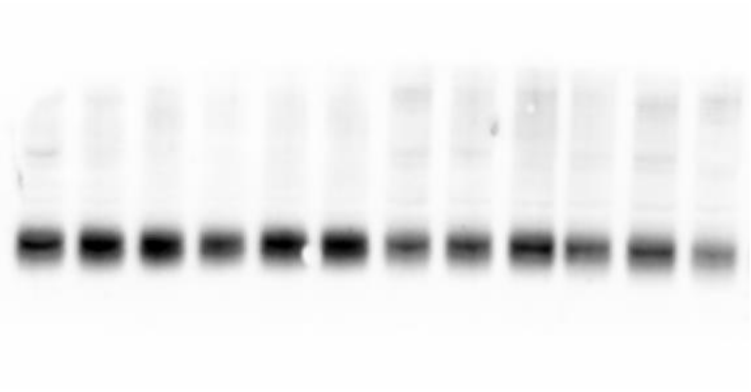

Blot 2

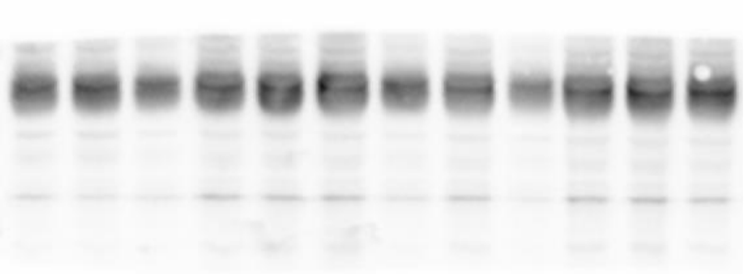

GLUT1:

Blot 1

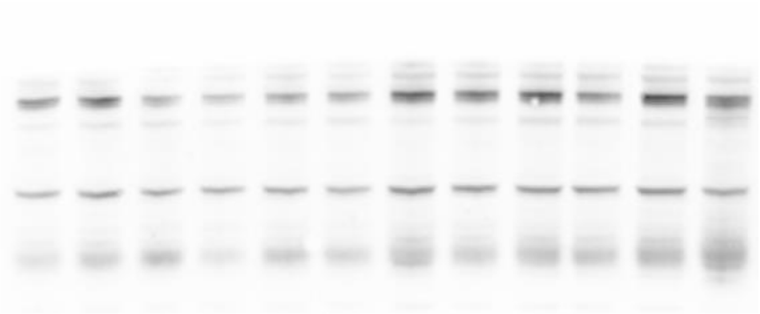

Blot 2

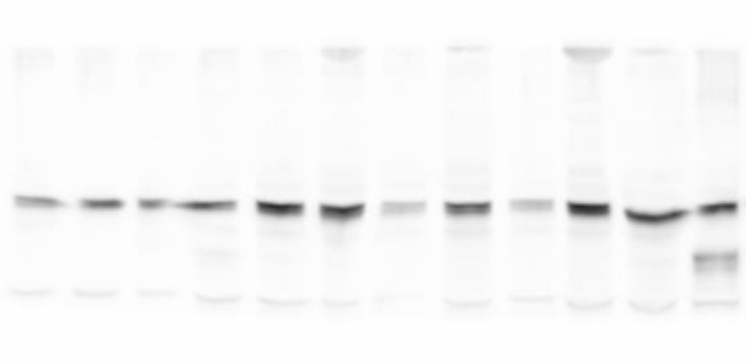

LDH:

Blot 1

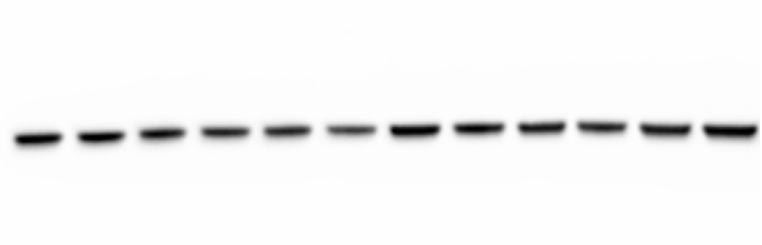

Blot 2

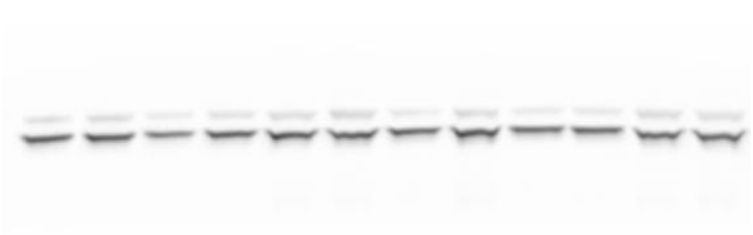

PDH:

Blot 1:

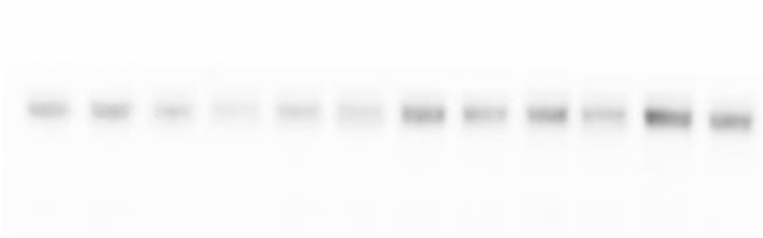

Blot 2

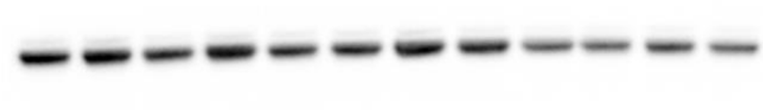

PDH p232:

Blot 1

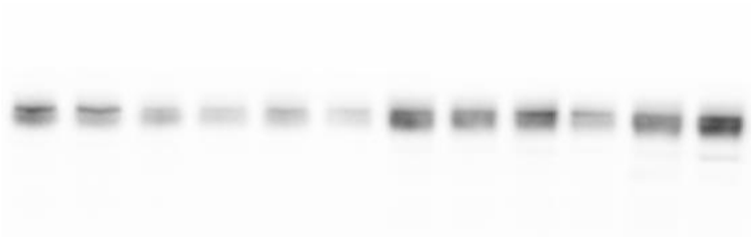

Blot 2

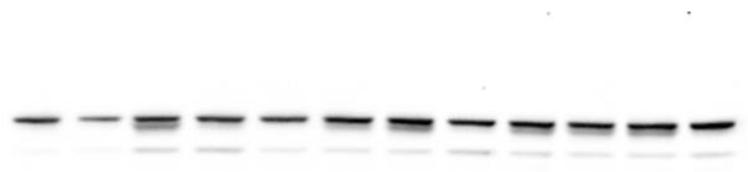

PDH p293:

Blot 1

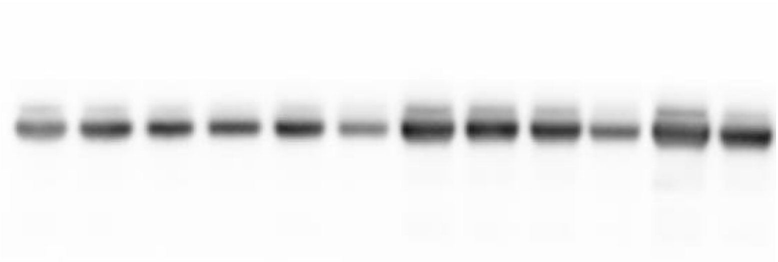

Blot 2

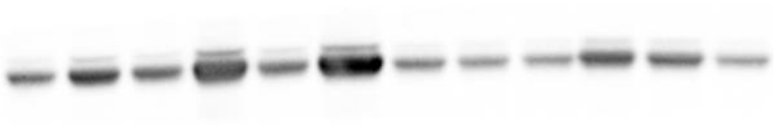

PDH p300:

Blot 1

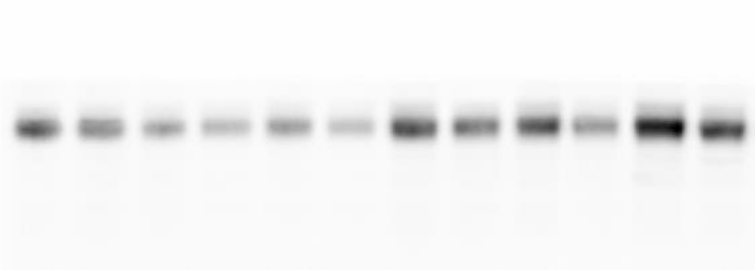

Blot 2

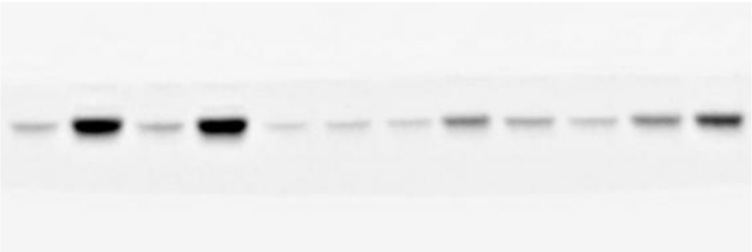

CD36:

Blot 1

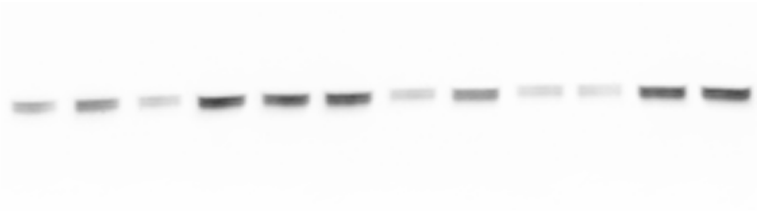

Blot 2

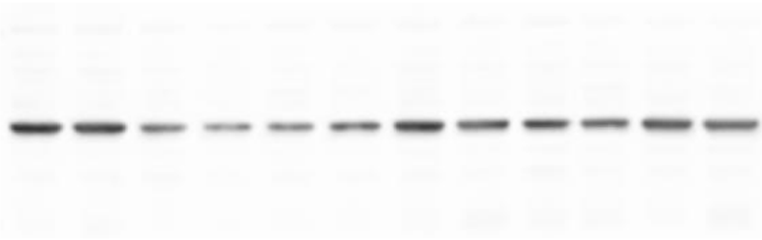

CPT1b:

Blot 1

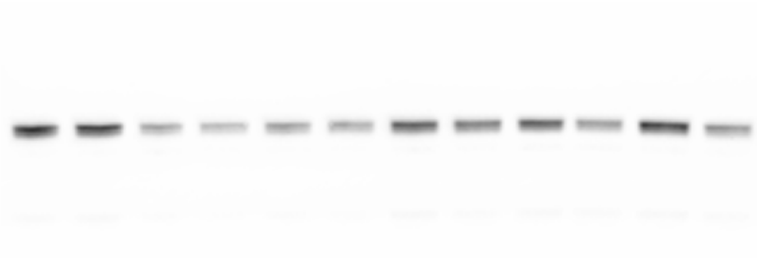

Blot 2

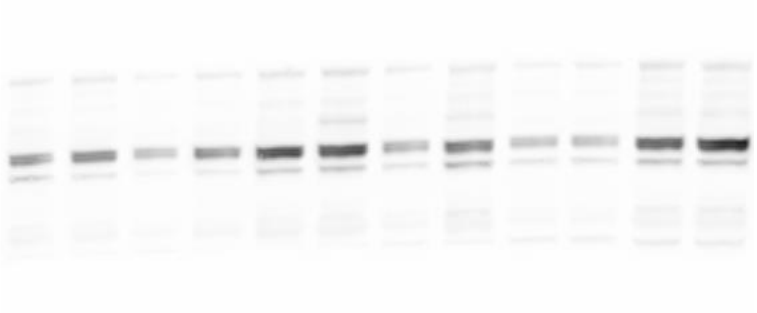

CD68:

Blot 1

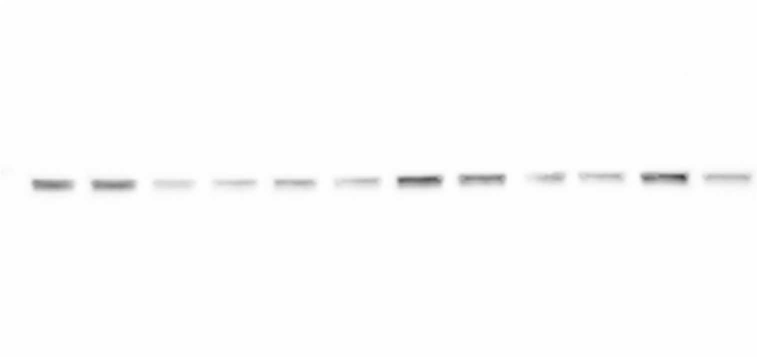

Blot 2

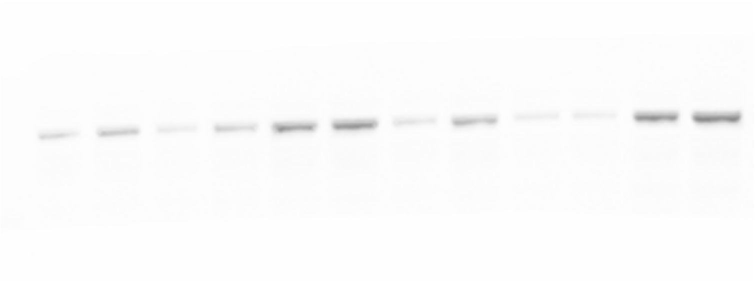

Collagen:

Blot 1

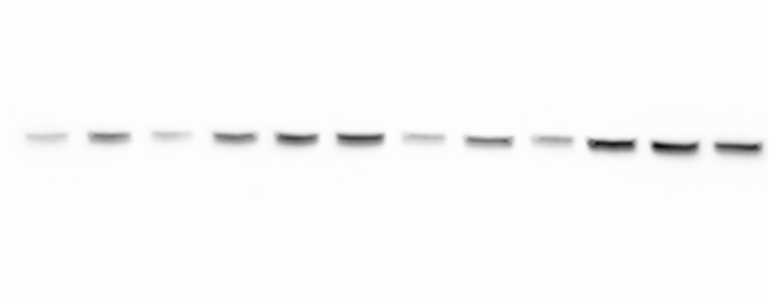

Blot 2

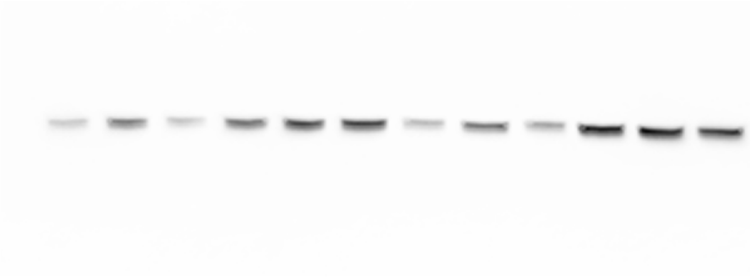

ASkMA:

Blot 1

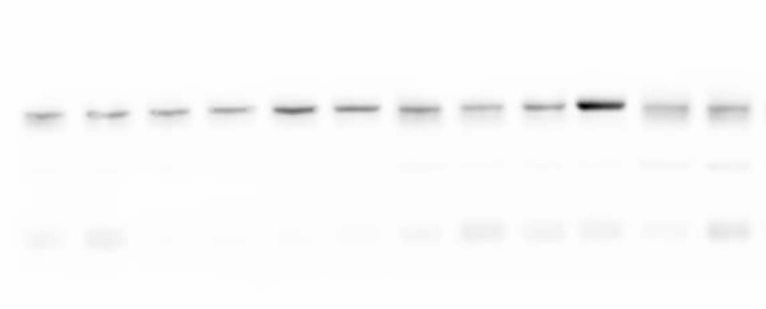

Blot 2

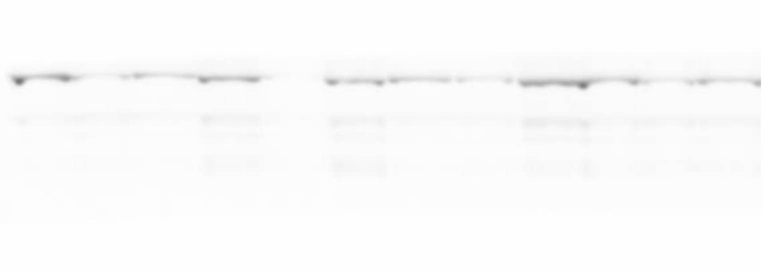

Supplement: cvab081_Supplementary_Data [file cvab081_supplementary_data.pdf]
